# Nationwide population-based infection- and vaccine-induced SARS-CoV-2 antibody seroprevalence in Germany in autumn/winter 2021/2022

**DOI:** 10.2807/1560-7917.ES.2025.30.1.2400037

**Published:** 2025-01-09

**Authors:** Elisabetta Mercuri, Lorenz Schmid, Christina Poethko-Müller, Martin Schlaud, Cânâ Kußmaul, Ana Ordonez-Cruickshank, Sebastian Haller, Ute Rexroth, Osamah Hamouda, Lars Schaade, Lothar H Wieler, Antje Gößwald, Angelika Schaffrath Rosario, on behalf of the RKI-SOEP-2 Study Group

**Affiliations:** 1 Robert Koch Institute (RKI), Berlin, Germany; 2 Max Planck Institute for Infection Biology, Berlin, Germany; 3 Digital Global Public Health at the Hasso-Plattner-Institute (HPI), University Potsdam, Potsdam, Germany; 4 RKI-SOEP-2 Study Group is acknowledged at the end of the article

**Keywords:** seroprevalence, SARS-CoV-2, antibody, vaccination, underreporting, public health surveillance

## Abstract

**Background:**

The first Corona Monitoring Nationwide (RKI-SOEP) study (October 2020−February 2021) found a low pre-vaccine SARS-CoV-2 antibody seroprevalence (2.1%) in the German adult population (≥ 18 years).

**Aim:**

The objective of this second RKI-SOEP (RKI-SOEP-2) study in November 2021−March 2022 was to estimate the prevalence of SARS-CoV-2-specific anti-spike and/or anti-nucleocapsid (anti-N) IgG antibodies (combined seroprevalence), past infection based on infection-induced seroprevalence (anti-N), and basic immunisation (at least two antigen contacts through vaccination or infection) in individuals aged ≥ 14 years. We also aimed to estimate under-reporting of infections.

**Methods:**

Dried blood-spot specimens from a population-based sample embedded in a dynamic cohort, the Socio-Economic Panel (SOEP), were serologically analysed. Resulting serological data and self-reports via a questionnaire from the same individuals were used to estimate prevalences.

**Results:**

Combined seroprevalence was 90.7% (95% CI: 89.7%–91.6%) without correction and 94.6% (95% CI: 93.6%–95.7%) with correction for sensitivity/specificity and antibody waning. While one in nine individuals had been infected (11.3%; 95% CI: 9.1%–13.5%), nine in 10 had a basic immunisation (90%; 95% CI: 88.9–90.9%), primarily due to vaccination. Population-weighted estimates differed by age, region, and socioeconomic deprivation. The under-reporting factor was estimated as 1.55 (95% CI: 1.3–1.8).

**Conclusions:**

When the SARS-CoV-2-Omicron wave was beginning, most people had been vaccinated, infected, or both. Large-scale vaccination, but not a high infection rate, was able to fill the immunity gap, especially in ≥ 65 year-olds who are known to be at higher risk of severe COVID-19. Our data point towards the need for targeted socioeconomically, demographically and regionally stratified mitigation strategies, including measures to enhance vaccine uptake.

**Key public health message**

**What did you want to address in this study and why?**

National surveillance data on SARS-CoV-2 infections and COVID-19 vaccinations may be incomplete and are often not available for subgroups of the population. Therefore, between November 2021 and March 2022, we performed a nationwide study based on antibody analysis and questionnaire information to estimate the proportion of the population in Germany that had been in contact with SARS-CoV-2 through infection and/or vaccination.

**What have we learnt from this study?**

When the Omicron wave was beginning, 19 of 20 people had already been exposed at least once to SARS-CoV-2 antigens. One in nine individuals had been infected with SARS-CoV-2, and nine in 10 had basic immunisation, primarily from vaccination and to a much lesser extent from vaccination and infection combined. Exposure to SARS-CoV-2 differed with age, region, and socioeconomic status of the district of residence. Only ca one in three infections had not been notified in the first 2 pandemic years.

**What are the implications of your findings for public health?**

Our results show how large-scale vaccination, but not a high infection rate, was able to provide basic immunisation to the population, especially for people aged ≥ 65 years who are known to be at higher risk of severe COVID-19. Moreover, it was found that the COVID-19 notification system yielded reliable data with only moderate under-reporting. The study results also highlight the need for targeted socioeconomically, demographically and regionally stratified prevention and vaccination strategies.

## Introduction

National surveillance data give a good overview on known severe acute respiratory syndrome coronavirus 2 (SARS-CoV-2) infections and overall vaccination coverage. However, uncertainty remains about the true exposure state of the population, especially given the unknown proportion of infections with mild or asymptomatic course that are not notified [1]. Moreover, vaccination data are only available at aggregate levels in Germany, leading to uncertainty about population hybrid immunity, which arises from the combination of infection and vaccination and has been found to offer the highest magnitude and durability of protection [2-5].

**Figure 1:**
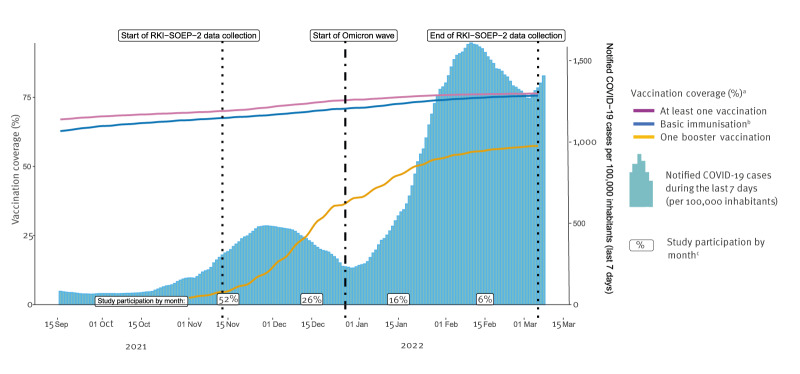
Vaccination coverage and notified basic immunisation^a^ in the country, as well as notified COVID-19 cases during the last 7 days per 100,000 inhabitants, and monthly percentage of participants enrolled in the RKI-SOEP-2 study, Germany, September 2021–March 2022

DIM: Digital Vaccination Coverage Monitoring; RKI-SOEP-2: second Corona Monitoring Nationwide study based on a population sample embedded in a dynamic cohort (the Socio-Economic Panel; SOEP).

^a^ Vaccination coverage and basic immunisation data were from the national surveillance (COVID-19 DIM), including those for children aged 5–11 years, for whom vaccination was not generally recommended until December 2021, and adolescents aged 12–17 years, for whom vaccination was not generally recommended until August 2021; the age-group-specific vaccination coverage is shown in Supplement 1.

^b^ Basic immunisation was defined as two vaccinations, or an infection followed by at least one vaccination. Note that in the DIM, there is less information available for defining basic immunisation than in the RKI-SOEP-2 study, the major difference being that the assessment of vaccination with Jcovden (previously COVID-19 Vaccine Janssen) changed over time and that no antibody results are available in the DIM. Moreover, only infections that occurred before vaccination (not afterwards) can be counted in the DIM. Supplement 7.1 provides more details.

^c^ Percentage of participants enrolled in the RKI-SOEP-2 study per month, population-weighted (n = 11,162; white boxes).

In Germany, the first Corona Monitoring Nationwide (RKI-SOEP) Study conducted from October 2020 to February 2021 (but predominantly in October/November), had shown that shortly before the start of the immunisation programme, only 2.1% of adults (≥ 18 years old) had been infected with SARS-CoV-2, indicating an effective containment strategy [6]. About half of these cases had been notified [6]. The second RKI-SOEP (RKI-SOEP-2) study, presented here, was conducted from November 2021 to the beginning of March 2022 during the fourth pandemic wave, dominated by the Delta variant, followed by the Omicron wave beginning around the turn of the year 2021–22 [7] (Figure 1). The vaccination campaign had been running for a year, and the booster campaign had been initiated [8] (Figure 1); the vaccination coverage by age group is presented in Supplement 1. In November 2021, the ‘2G’ rule had been implemented which allowed only people who had either recovered from a SARS-CoV-2 infection (i.e. had had a PCR-confirmed infection) or were fully vaccinated (two vaccinations or the combination of vaccination and infection) to enter or attend certain facilities or events (*‘G’* for the German words *‘genesen’* and ‘*geimpft’*, i.e. ‘recovered’ and ‘vaccinated’). In January 2022, the ‘2G plus’ rule added the requirement of an up-to-date negative antigen test.

Data sources for vaccination coverage and COVID-19 cases were accessed on 11 September 2023 (https://github.com/orgs/robert-koch-institut/repositories).

The aims of the study were to estimate the population-weighted prevalence, overall and stratified, of (i) being seropositive for anti-SARS-CoV-2 IgG antibodies targeting the nucleocapsid and the spike protein, (ii) having been infected with SARS-CoV-2, (iii) having a basic immunisation from at least two exposures to SARS-CoV-2 (from two or more doses of vaccine, or the combination of vaccination and infection i.e. hybrid immunity); and to estimate the under-reporting factor.

## Methods

### Study design and study population

We present cross-sectional results of the second Corona Monitoring Nationwide study (RKI-SOEP-2), a cooperative project of the Robert Koch Institute (RKI, the national public health institute in Germany), the Socio-Economic Panel (SOEP) at the German Institute for Economic Research (DIW Berlin), the Institute for Employment Research (IAB), and the Research Center of the Federal Office for Migration and Refugees (BAMF-FZ). The study was embedded in the Socio-Economic Panel, a nationwide dynamic cohort built upon population-based random samples, and comprised individuals aged ≥ 14 years from the 2021 SOEP study. The study protocol has been published elsewhere [9]. SOEP households were invited by postal mail. Participants received a post-paid monetary incentive (EUR 10 for adults aged ≥ 18 years, EUR 5 for adolescents aged 14−17 years) as well as a written laboratory test result notification for their personal information.

### Data collection and laboratory methods

Data collection began on 13 November 2021 and ended on 6 March 2022. Study materials included a questionnaire of 12 pages (on paper or online), a disposable kit for the self-sampling of capillary blood from a finger prick, illustrated instructions and a link to corresponding video instructions. The questionnaire (available in seven languages), which could be self-completed by study participants regardless of their age, included questions on past SARS-CoV-2 infection (known PCR-confirmed infection, test date, disease severity), vaccination status (number of doses, date and site of vaccination, vaccine type), attitudes towards the pandemic, health status, and health behaviours [9].

Participants sent dried blood samples (DBS) by mail to the RKI laboratory. SARS-CoV-2 immunoglobulin G (IgG) antibodies against the S1 domain of the spike protein (anti-S) were assessed quantitatively, and anti-nucleocapsid protein IgG antibodies (anti-N) with a semiquantitative assay (Euroimmun AG, Lubeck, Germany; see also Supplement 2.1). The cutoff for anti-N seropositivity was adapted from the manufacturer-supplied value (1.1) to account for the use of DBS and set to 0.95, while the cutoff ≥ 35.2 Binding Antibody Units (BAU)/mL for anti-S seropositivity remained unchanged, following results of an internal validation study presented in Supplement 2.2.

### Primary outcomes

A detailed description of the indicators and handling of missing values is provided in Supplement 3 and Supplementary Figure S3.

*Combined seroprevalence* was defined as seropositivity for anti-N and/or anti-S antibodies. We present seroprevalence estimates both without and with correction for test characteristics. The correction also accounts for antibody waning (as described in the analysis methods below) and thus allows estimation of the proportion of the population that has ever been exposed to SARS-CoV-2 antigens.

*Infection-induced seroprevalence*, defined as seropositivity for anti-N antibodies, with correction for test characteristics and antibody waning over time, was our main indicator for past SARS-CoV-2 infection. In contrast to ‘infection status’, this outcome accounts for previously undetected infections. For means of comparison, we also present uncorrected seroprevalence estimates.

*SARS-CoV-2 infection status* was defined as ‘past infection’ when having any of self-reported infection (known PCR-confirmed infection), seropositivity for anti-N antibodies or, if self-reported as unvaccinated, for anti-S antibodies. It was not corrected for test characteristics and antibody waning. We had used this indicator as main outcome in preliminary results [10,11], as it is easy to analyse. Even without correction, it does account for known previous infections, but in cases where antibody waning has occurred i.e. no anti-N antibodies are present, unknown infections are not accounted for and thus a simple uncorrected analysis leads to lower prevalence estimates than infection-induced seroprevalence. Results with correction were very similar to corrected infection-induced seroprevalence and are thus not shown.

*Under-reporting* of infections in national COVID-19 surveillance data was estimated by linking the study data to surveillance data on an individual basis (by date, age, sex and district) as described in Supplement 4. The under-reporting factor was calculated as the ratio of corrected infection-induced seroprevalence to the prevalence of notified cases.

*Basic immunisation* was defined as having at least two antigen exposures, i.e. at least two self-reported vaccination doses, or a combination of at least one self-reported vaccination and an infection (self-reported or anti-N seropositivity). Regardless of their chronological order, we refer to the combination of natural infection and vaccination as ‘hybrid immunity’. A minimum time interval was required for two subsequent events to be counted as separate immunologically effective events. Basic immunisation was not corrected for test characteristics and antibody waning, since the corrected prevalence estimates were similar to the uncorrected ones (data not shown). The prevalence of hybrid immunity, however, was corrected.

We do not report on three or more antigen exposures here since the vaccination booster campaign was ongoing during field time.

### Covariable definitions

Results are presented stratified by age group (14–17, 18–34, 35–49, 50–64, 65–99 years), sex (collected as male/female/diverse and presented as male/female, with random allocation of the ‘diverse’ group), and place of residence. The latter was classified, firstly, into three categories of district-level socioeconomic deprivation (low: quintile 1, medium: quintiles 2–4, high: quintile 5), and secondly, into four regions based on the federal states. Instead of using individual socioeconomic status, which is not available in notification data needed for the estimation of under-reporting, we used area-level socioeconomic deprivation to account for socioeconomic disparities. This was measured at the level of Germany’s 400 districts using the German Index of Socioeconomic Deprivation (GISD) [12,13], which measures relative deprivation in the domains of education, employment and income. Federal states were grouped into an eastern (Berlin, Brandenburg, Saxony, Saxony-Anhalt, Thuringia), western (North Rhine-Westphalia, Hesse, Rhineland-Palatinate, Saarland), southern (Bavaria, Baden-Württemberg) and northern region (Schleswig-Holstein, Bremen, Hamburg, Lower Saxony, Mecklenburg-Pomerania), reflecting the geography as well as the pattern of notified infections over the course of the pandemic. A map and population sizes as well as an analysis by federal state are presented in Supplement 11.

### Statistical analyses

All analyses were weighted to allow generalisation to the general population and to adjust for survey non-response as well as panel attrition. The weighting factors resulted from complex modelling of contact ability and participation probabilities at both the household and the individual level. Weights were adjusted at the household (household type, size, home ownership, and federal state) and the individual level (age, sex, and German citizenship) to match population distributions [9,14,15].

Descriptive results are presented as unweighted numbers and population-weighted percentages, with stratification by the four following variables: sex, age group, district-level socioeconomic deprivation and region. Missing data were handled by available-case analysis. In analyses without correction for test characteristics and antibody waning, 95% confidence intervals (CI) were calculated as Wald intervals on the logit scale, based on robust standard errors calculated via survey procedures to account for weighting and household clustering. Logistic regression models were estimated with survey procedures to obtain adjusted odds ratios (OR), with the four stratification variables and the month of study participation (categorical) as covariates. Based on the estimated ORs, we calculated model-adjusted prevalences with the Stata ‘margins’ command and 95% logit CIs with the command ‘coef_table’. The model-adjusted prevalences are obtained by predicting the probability of being positive from the model, thus adjusting for the covariates, and averaging these predicted probabilities within each stratum. Analyses were performed with Stata 16.1 (StataCorp, 2019, College Station, Texas, United States) and SAS 9.4 (SAS Institute Inc., Cary, North Carolina, United States).

### Correction for test characteristics and antibody waning

Combined seroprevalence, infection-induced seroprevalence and hybrid immunity were corrected for test characteristics (specificity and sensitivity, including antibody waning).

For specificity, we used values provided by the Paul Ehrlich Institute [16]: 99.4% (672/676; 95% CI: 98.5–99.8) for the anti-S assay and 99.3% (671/676; 95% CI: 98.3–99.8) for the anti-N assay. For combined seroprevalence, we used anti-S specificity, as the proportion of participants who were anti-S seronegative but anti-N seropositive was small (28/10,342 = 0.3%).

Sensitivity was estimated internally from the study population in order to account for antibody waning, i.e. we estimated (i) the proportion that was seropositive for combined seroprevalence (95.8%; 95% CI: 95.1–96.4) among participants with a self-reported vaccination or a self-reported positive SARS-CoV-2 test at least 11 days pre-study (n = 9,260); and (ii), the proportion that was seropositive for infection-induced seroprevalence (47.4%; 95% CI: 41.6–53.3) among participants with a self-reported positive SARS-CoV-2 test at least 11 days pre-study (n = 774). For stratified analyses, we estimated sensitivity within the same strata that were used for the analysis as described in Supplement 5.

Using external sensitivity estimates is problematic, since factors affecting antibody waning (such as time since infection/vaccination, age, virus variant, severity of disease) may differ between the study population and the external clinical sample used for determining sensitivity. By using internal estimates, and by estimating sensitivity within the same strata used for analysis, we could ensure as far as possible that the mixture of these factors is similar in the subgroup used for estimating sensitivity and the entire study population. Note that this does not necessarily imply that sensitivity is homogeneous within strata.

In stratified analyses of seroprevalence, within each stratum we applied the usual formula [17] for the correction:

${prevalence}_{corrected}=\frac{{prevalence}_{observed}+specificity-1}{sensitivity+specificity-1}$ ,

using stratum-specific sensitivity estimates. We calculated 95% CIs as described in Supplement 6.1, i.e. taking into account that sensitivity and specificity were estimated and thus subject to random variability [17].

In logistic regression and for the composite variable ‘hybrid immunity’, we corrected for test characteristics and antibody waning using predictive value weighting [18], with 95% bootstrap CIs taking account of household clustering and the random uncertainty in sensitivity and specificity [19], as described in the Supplement’s subsections 6.2 and 6.3.

## Results

The study included 11,162 participants aged 14–99 years from 6,760 households, out of 20,774 invited individuals from 11,785 households (response 53.7% [9]; drop-out reasons are presented in Supplement 9). A total of 10,687 participants (95.7%) had DBS specimens yielding valid laboratory results. Valid questionnaires were available for 10,985 participants (98.4%). Questionnaire items on prior vaccination and known infection were answered by 10,884 participants (97.5%; all percentages in this paragraph unweighted).

**Figure 2:**
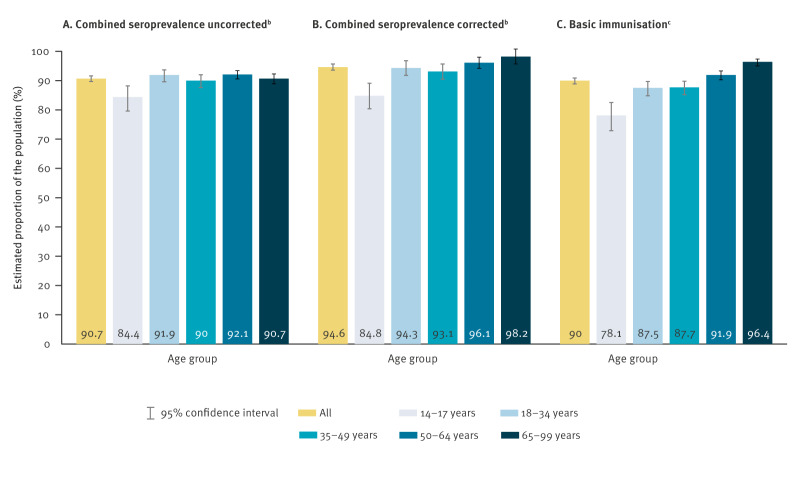
Estimated combined SARS-CoV-2 IgG antibody seroprevalence (A) without and (B) with correction for test characteristics and antibody waning (n = 10,687) and (C) estimated basic immunisation prevalence (n = 10,932) in community-dwelling people (≥ 14 years) stratified by age group, Germany (RKI-SOEP-2 study), autumn/winter 2021/22^a^

RKI-SOEP-2: second Corona Monitoring Nationwide study based on a population sample embedded in a dynamic cohort (the Socio-Economic Panel; SOEP); SARS-CoV-2: severe acute respiratory syndrome coronavirus 2.

^a^ The RKI-SOEP-2 study collected data between 13 November 2021 and 6 March 2022 but these were predominantly obtained between November 2021 and January 2022.

^b^ More detail can be found in Table 1.

^c^ More detail can be found in Table 3.

In autumn/winter 2021/2022 in Germany in the 14–99-year age group, uncorrected combined seroprevalence was 90.7% (95% CI: 89.7%–91.6%; Table 1, Figure 2). Correcting the estimate for specificity and internally estimated sensitivity increased the combined seroprevalence to 94.6% (95% CI: 93.6%–95.7%). Corrected combined seroprevalence was lowest in the youngest age group (14–17 years: 84.8%; 95% CI: 80.4%–89.1%) and in the eastern German region (89.5%; 95% CI: 86.7%–92.3%). It was highest in the older age groups (50–64 years: 96.1%; 95% CI: 94.2%–98.0%, 65–99 years: 98.2%; 95% CI: 95.7%–100.8%) and in areas with low socioeconomic deprivation (96.5%; 95% CI: 94.6%–98.5%).

**Table 1:** Estimates, based on study participants with valid dried blood spot specimens, of combined seroprevalence of SARS-CoV-2 anti-spike and anti-nucleocapsid IgGs in community-dwelling people (≥ 14 years), stratified across demographic and socio-geographic characteristics, Germany (RKI-SOEP-2 study), autumn/winter 2021/22 (n = 10,687 participants)^a^

Characteristic	Participants with combined seroprevalence information^b^		Participants N+^c^		Participants S+^d^		Participants N+ and/or S+	Uncorrected combined seroprevalence (N+ and/or S+) %^g^ (95% CI)	p	Corrected^e^ combined seroprevalence (N+ and/or S+) % (95% CI)	Model-adjusted^f^ OR (95% bootstrap CI)	Model-adjusted^f^ and corrected combined seroprevalence (N+ and/or S+) % (95% bootstrap CI)
	Number	Column %	Number	%^g^	Number	%^g^	Number					
All 14–99-yr-olds	10,687	100	621	6.0	9,767	90.4	9,795	90.7 (89.7–91.6)	NA	94.6 (93.6–95.7)	NA	NA
All 18–99-yr-olds	10,047	100	568	5.8	9,227	90.9	9,253	91.2 (90.3–92.1)		95.5 (94.4–96.6)	NA	NA
**Sex**												
Women	5,768	51.1	331	5.3	5,284	90.6	5,300	90.8 (89.6–91.9)	0.76	94.3 (92.8–95.7)	Ref.	94.1 (92.9–95.1)
Men	4,919	48.9	290	6.7	4,483	90.2	4,495	90.6 (89.2–91.8)		95.0 (93.3–96.8)	1.30 (0.96–1.78)	95.3 (94.2–96.3)
**Age group**												
14–17 yrs	640	7.9	53	8.2	540	84.3	542	84.4 (79.6–88.2)	0.0007	84.8 (80.4–89.1)	0.34 (0.21–0.53)	83.9 (79.4–88.0)
18–34 yrs	1,869	22.6	113	6.2	1,721	91.3	1,727	91.9 (89.6–93.7)		94.3 (91.8–96.8)	Ref.	93.7 (91.9–95.4)
35–49 yrs	2,387	20.8	163	7.9	2,168	89.7	2,177	90.0 (87.6–92.0)		93.1 (90.5–95.7)	0.92 (0.60–1.43)	93.2 (91.2–95.0)
50–64 yrs	3,387	25.6	177	4.5	3,136	92.1	3,142	92.1 (90.6–93.4)		96.1 (94.2–98.0)	1.78 (1.12–2.75)	96.3 (95.2–97.4)
65–99 yrs	2,404	23.0	115	4.7	2,202	90.5	2,207	90.7 (88.9–92.3)		98.2 (95.7–100.8)	4.22 (2.23–6.94)	98.4 (97.4–99.0)
**Socioeconomic deprivation^h^**												
Low	2,585	25.9	140	6.3	2,403	92.6	2,414	93.1 (91.4–94.5)	0.0005	96.5 (94.6–98.5)	Ref.	96.5 (94.6–97.9)
Medium	6,515	60.0	381	5.6	5,965	90.3	5,978	90.4 (89.1–91.6)		94.1 (92.6–95.7)	0.59 (0.32–1.06)	94.4 (93.2–95.4)
High	1,487	14.1	95	6.9	1,306	86.8	1,310	87.1 (83.9–89.7)		93.3 (89.4–97.2)	0.46 (0.21–0.96)	92.9 (89.6–95.4)
**Region**												
Northern	1,960	17.8	77	3.8	1,810	89.9	1,812	89.9 (87.3–92.1)	< 0.0001	95.1 (91.8–98.5)	0.58 (0.35–1.12)	95.0 (93.0–96.9)
Western	3,368	34.7	165	5.3	3,143	92.2	3,145	92.3 (90.6–93.6)		96.6 (94.6–98.5)	Ref.	97.0 (95.7–97.9)
Southern	3,001	29.5	177	6.4	2,749	91.4	2,763	92.0 (90.3–93.5)		95.3 (93.4–97.3)	0.54 (0.28–1.02)	94.6 (92.0–96.4)
Eastern	2,358	18.1	202	8.8	2,065	86.0	2,075	86.3 (83.6–88.7)		89.5 (86.7–92.3)	0.29 (0.18–0.47)	90.6 (88.2–92.9)

Anti-N: anti-nucleocapsid; anti-S: anti-spike; CI: confidence interval; IgG: immunoglobulin G; NA: not applicable; N+: anti-N IgG seropositive; OR: odds ratios; ref.: reference; RKI-SOEP-2: second Corona Monitoring Nationwide study based on a population sample embedded in a dynamic cohort (the Socio-Economic Panel; SOEP); SARS-CoV-2: severe acute respiratory syndrome coronavirus 2; S+: anti-S IgG seropositive; yr: year.

^a^ The RKI-SOEP-2 study collected data between 13 November 2021 and 6 March 2022 but these were predominantly obtained between November 2021 and January 2022.

^b^ Participants with combined seroprevalence information are those with a dried blood spot specimen yielding a valid laboratory result for anti-N or anti-S IgGs. In practice, these were the same as participants with a valid anti-S IgG result (see footnotes c and d). There were 10,687 participants with combined seroprevalence information.

^c^ The percentages of N+ are based on the number of participants with available anti-N IgG results, which was 10,342. This is because not all blood spots provided by the participants had enough material to conduct both the anti-N and anti-S IgG tests; hence among 10,687 blood spots, all were analysed by anti-S IgG test but only 10,342 by anti-N IgG test. The stratification of study participants with anti-N IgG tests across sex, age group, socioeconomic deprivation and region is shown in Table 2.

^d^ The percentages of S+ are based on the number of participants with available anti-S IgG results, which was 10,687. Their numbers stratified across sex, age group, socioeconomic deprivation and region are the same as those of participants with combined seroprevalence information.

^e^ The corrected seroprevalences for ‘N+ and/or S+’ are corrected for test characteristics (including anti-S specificity = 0.994) and stratum-specific antibody waning, as detailed in Supplement 5.1 and 6.1.

^f^ The model-adjusted ORs and seroprevalences are mutually adjusted for the variables in the table and month of study participation (as categorical variable). They are based on the 10,587 participants for whom information on all variables and study participation month was available (see footnote h). The correction for test characteristics (including anti-S specificity = 0.994) and antibody waning was performed by using predictive value weights when fitting the model, as described in Supplement 6.2.

^g^ Percentage without correction for test characteristics and antibody waning.

^h^ Numbers do not add up to a total of 10,687 due to missing data concerning the socioeconomic deprivation for 100 participants (available-case analysis).

All percentages are population-weighted. All analyses are for 14–99-year-olds unless otherwise specified.

Data for the figure were obtained by the RKI-SOEP-2 study.

All percentages are weighted to match population distributions. The prevalence of basic immunisation is not corrected for test characteristics and antibody waning, since the correction leads to similar results (data not shown).

To estimate the prevalence of infection status, three groups of participants were considered: (i) those with a self-report on prior PCR-test confirmed SARS-CoV-2 infection (n = 10,889), (ii) those with a valid test on SARS-CoV-2 anti-N antibodies (n = 10,342) and (iii) those with information on their vaccination status and who also had a valid anti-S antibody test (n = 10,501). Altogether, the number of participants belonging to at least one of these groups was 11,154. The stratified numbers and proportions of participants with evidence of prior infection within each respective group are shown in Supplement 8.

**Figure 3:**
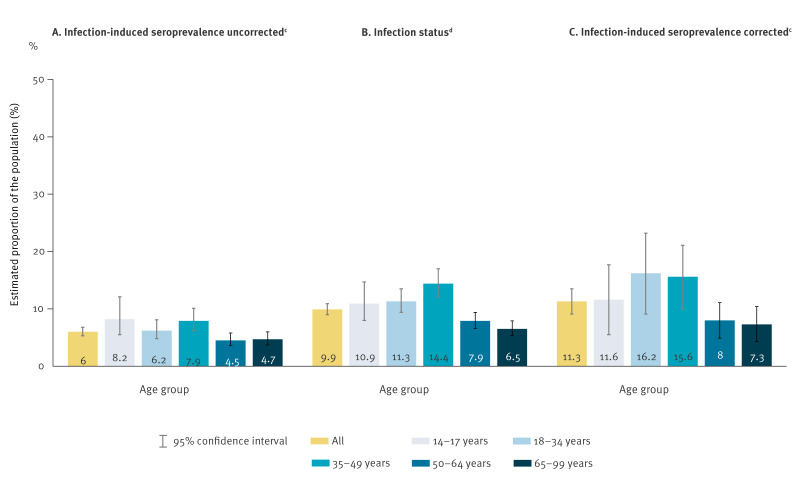
(A) SARS-CoV-2-infection-induced seroprevalence estimated without correction^a^ (n = 10,342), as well as (B) estimated infection status (n = 11,154) and (C) infection-induced seroprevalence estimated with correction^a^ (n = 10,342) among community-dwelling people (≥ 14 years) stratified by age group, Germany (RKI-SOEP-2 study), autumn/winter 2021/22^b^

Anti-N: anti-nucleocapsid IgG; RKI-SOEP-2: second Corona Monitoring Nationwide study based on a population sample embedded in a dynamic cohort (the Socio-Economic Panel; SOEP); SARS-CoV-2: severe acute respiratory syndrome coronavirus 2.

^a^ The infection-induced seroprevalence was estimated based on anti-N seroprevalence. It is presented as (A) uncorrected and (C) corrected for test characteristics and antibody waning.

^b^ The RKI-SOEP-2 study collected data between 13 November 2021 and 6 March 2022 but these were predominantly obtained between November 2021 and January 2022.

^c^ More detail can be found in Table 2.

^d^ Infection status is based on self-reported infections as well as uncorrected anti-N seroprevalence and, in non-vaccinated individuals, anti-S antibody seroprevalence. More detail can be found in Table 2.

Estimates of proportions of the population with past SARS-CoV-2 infection based on infection status and infection-induced (anti-N) seroprevalence are shown in Table 2. The proportion of the population with past SARS-CoV-2 infection was estimated as 11.3% (95% CI: 9.1%–13.5%) based on the corrected infection-induced (anti-N) seroprevalence (Table 2, Figure 3). This estimate was higher than that based on infection status, i.e. uncorrected antibody analysis combined with self-reported known infections (9.9%; 95% CI: 9.0%–10.9%), and almost twice as high as the estimate using uncorrected anti-N antibodies only (6%; 95% CI: 5.3%–6.8%), due to the rather low sensitivity of the antibody test. Infection-induced seroprevalence first increased with age, with maxima of 16.2% and 15.6% in the age groups 18–34 and 35–49 years (with correction; 95% CI: 9.1%–23.2% and 10%–21.1%), but was only half as high in the higher age groups (8%; 95% CI: 4.9%–11.1% for 50–64 years and 7.3% for 65–99 years; 95% CI: 4.3%–10.4%). It was highest in the eastern region (16.7%; 95% CI: 11.2%–22.2%) and lowest in the northern region (7.1%; 95% CI: 3.1%–11.2%). The observed patterns for infection status were similar, the exception being a lower prevalence estimate in the age group 18–34 years compared with 35–49 years.

**Table 2:** Estimates in community-dwelling people (≥ 14 years) of SARS-CoV-2 infection prevalence, based on infection status and infection-induced seroprevalence, as well as estimation of infection under-reporting, stratified across demographic and socio-geographic characteristics, Germany (RKI-SOEP-2 study), autumn/winter 2021/22 (n = 11,154 participants)^a^

Characteristic	Participants with information to determine infection status		Participants with prior infection, based on infection status^b^		Participants with valid anti-N test	Participants N+	Corrected infection-induced seroprevalence^c^ (N+), % (95% CI)	Notified cases %^d^	Under-reporting factor^e^	p	Model-adjusted^f^ OR (95% bootstrap CI)	Model-adjusted^f^ and corrected infection-induced seroprevalence (N+) % (95% bootstrap CI)
	Number	Column %	Number	%^g^ (95% CI)	Number	Number						
All 14–99-yr-olds	11,154	100	1,193	9.9 (9.0–10.9)	10,342	621	11.3 (9.1−13.5)	7.3	1.55 (1.3−1.8)	NA	NA	NA
All 18–99-yr-olds	10,442	100	1,100	9.8 (8.9–10.8)	9,729	568	11.4 (9.1−13.6)	7.0	1.62 (1.3−1.9)	NA	NA	NA
**Sex**												
Women	5,996	50.9	651	9.1 (8.1–10.2)	5,603	331	10.2 (7.4−13.0)	7.3	1.39 (1.0−1.8)	Ref.	Ref.	10.9 (8.6–14.5)
Men	5,158	49.1	542	10.8 (9.5–12.2)	4,739	290	12.4 (9.0−15.9)	7.2	1.72 (1.2−2.2)	0.28	1.19 (0.87–1.66)	12.7 (10.1–16.6)
**Age group**												
14–17 yrs	712	8.4	93	10.9 (8.0–14.7)	613	53	11.6 (5.5−17.7)	10.3	1.12 (0.5−1.7)	0.27	0.71 (0.35–1.26)	11.6 (7.4–17.1)
18–34 yrs	1,932	22.3	241	11.3 (9.4–13.5)	1,814	113	16.2 (9.1−23.2)	9.8	1.65 (0.9−2.4)	Ref.	Ref.	15.5 (10.7–24.2)
35–49 yrs	2,477	20.6	329	14.4 (12.1–17.0)	2,350	163	15.6 (10.0−21.1)	8.8	1.77 (1.1−2.4)	0.81	1.04 (0.58–1.74)	16.0 (12.5–21.2)
50–64 yrs	3,487	25.4	339	7.9 (6.6–9.4)	3,293	177	8.0 (4.9−11.1)	6.1	1.30 (0.8−1.8)	0.42	0.46 (0.25–0.75)	7.9 (5.3–10.6)
65–99 yrs	2,546	23.3	191	6.5 (5.4–7.9)	2,272	115	7.3 (4.3−10.4)	3.7	2.00 (1.2−2.8)	0.54	0.48 (0.25–0.83)	8.1 (5.7–11.7)
**Socioeconomic deprivation** ^h^												
Low	2,700	26.0	260	9.6 (7.8–11.8)	2,502	140	11.0 (6.5−15.5)	7.4	1.49 (0.9−2.1)	Ref.	Ref.	12.0 (8.5–17.4)
Medium	6,779	59.8	751	10.0 (8.9–11.3)	6,306	381	11.2 (8.2−14.2)	7.3	1.54 (1.1− 2.0)	0.89	0.97 (0.59–1.59)	11.7 (9.1–15.7)
High	1,572	14.3	171	9.6 (7.5–12.1)	1,438	95	10.6 (6.2−15.0)	7.2	1.47 (0.9−2.1)	0.96	0.93 (0.49–1.79)	11.3 (7.7–17.2)
**Region**												
Northern	2,030	17.7	130	6.3 (4.8–8.2)	1,910	77	7.1 (3.1−11.2)	4.6	1.55 (0.7−2.4)	0.95	0.80 (0.41–1.47)	8.4 (4.6–14.3)
Western	3,520	34.7	334	9.0 (7.6–10.7)	3,252	165	10.1 (6.3−14.0)	6.7	1.52 (0.9−2.1)	Ref.	Ref.	10.2 (7.5–14.2)
Southern	3,146	29.5	342	10.0 (8.4–12.0)	2,908	177	11.9 (7.5−16.3)	8.3	1.43 (0.9−2.0)	0.83	1.19 (0.74–1.94)	11.9 (8.4–17.3)
Eastern	2,458	18.0	387	15.0 (12.8–17.6)	2,272	202	16.7 (11.2−22.2)	9.5	1.76 (1.2−2.3)	0.56	1.90 (1.21–3.15)	17.5 (13.5–24.0)

Anti-N: anti-nucleocapsid IgG; CI: confidence interval; IgG: immunoglobulin G; NA: not applicable; N+: anti-N IgG seropositive; OR: odds ratio; Ref.: reference; RKI-SOEP-2: second Corona Monitoring Nationwide study based on a population sample embedded in a dynamic cohort (the Socio-Economic Panel; SOEP); SARS-CoV-2: severe acute respiratory syndrome coronavirus 2; yr: year.

^a^ The RKI-SOEP-2 study collected data between 13 November 2021 and 6 March 2022 but these were predominantly obtained between November 2021 and January 2022.

^b^ Based on self-reports of having tested positive by PCR for SARS-CoV-2 and uncorrected antibody results for anti-N and, for non-vaccinated participants, anti-spike antibodies.

^c^ Anti-N seroprevalence with correction for test characteristics (including specificity = 0.993) and antibody waning (see Supplement 5.2 and 6.1). This is our main estimate of the proportion of the population with past SARS-CoV-2 infection.

^d^ Cumulative proportion of notified cases, adjusted for sampling density (i.e. each participant contributes according to the sex-, age group- and district-specific cumulative proportion of notified cases with symptom onset corresponding to his/her dried blood spot testing date minus 14 days) and excluding deceased cases, as described in Supplement 4.

^e^ Under-reporting factor: ratio of infection-induced seroprevalence in the study to cumulative proportion of notified cases (see Supplement 4).

^f^ The model-adjusted ORs and seroprevalences are mutually adjusted for the variables in the table and month of study participation (as categorical variable). They are based on the 10,246 participants with available anti-N results for whom information on all variables and study participation month was also available (see footnote h). The correction for test characteristics (including anti-N specificity = 0.993) and antibody waning was performed by using predictive value weights when fitting the model, as described in Supplement 6.2.

^g^ Percentage without correction of antibody results for test characteristics and antibody waning. Infection status is shown for means of comparison and is not corrected for test characteristics and antibody waning. Correction led to results similar to corrected infection-induced seroprevalence (data not shown).

^h^ Numbers do not add up to a total of 11,154 due to missing values in the variable for socioeconomic deprivation for 103 participants (available-case analysis).

All percentages are population-weighted. All analyses for 14–99 years unless otherwise specified.

All percentages are population-weighted. Infection status is shown for means of comparison and is not corrected for test characteristics and antibody waning. Correction led to similar results as corrected infection-induced seroprevalence (data not shown).

We observed an overall under-reporting factor since the start of the pandemic of 1.55 (95% CI: 1.3–1.8) (Table 2). There was suggestive evidence of higher under-reporting in men compared with women and in the eastern region, while it seemed lower in adolescents, but no result reached statistical significance.

The prevalence of basic immunisation was estimated as 90.0% (95% CI: 88.9%–90.9%) without correction for test characteristics and waning (Table 3, Figure 2), and correction led to similar results (data not shown). The vast majority (88.1%; 95% CI: 87.0%–89.1%) received their basic immunisation from vaccination, 8.6% (with correction; 95% CI: 6.9%–10.3%) had hybrid immunity. Basic immunisation increased with age and decreased somewhat with higher socioeconomic deprivation. In the eastern region only 84.0% (95% CI: 81.0%–86.6%) of participants had a basic immunisation. Notably, basic immunisation was highest (96.4%; 95% CI: 95.0%–97.4%) in the age group with the lowest infection rate (65–99 years), and it was lowest in the (eastern) region with the highest infection rate. While infection and hybrid immunity (12.9%; 95% CI: 9.9%–16.5%) were highest in the eastern region, basic immunisation and the proportion who had received at least two doses of vaccine (80.8%; 95% CI: 77.6%–83.6%) was lowest there.

**Table 3:** Estimated basic immunisation status against SARS-CoV-2 in community-dwelling people (≥ 14 years), stratified across demographic and socio-geographic characteristics, Germany (RKI-SOEP-2 study), autumn/winter 2021/22 (n = 10,932 participants)^a^

Characteristics	Participants with information to determine basic immunisation		Participants with at least two vaccine doses (self-reported)		Hybrid immunity				Basic immunisation				
					Participants with information to determine hybrid immunity	Participants with hybrid immunity	Prevalence based on self-reports and uncorrected antibody results^b^	Prevalence based on self-reports and corrected antibody results^c^	Participants with basic immunisation	Prevalence based on self-reports and uncorrected^d^ antibody results	Model-adjusted^e^ OR (95% CI)	p^e^	Model-adjusted^e^ prevalence, % (95% CI)
	Number	Column %	Number	Prevalence % (95% CI)	Number	Number	%^b^ (95% CI)	%^c^ (95% bootstrap CI)	Number	%^d^ (95% CI)			
All 14–99-yr-olds	10,932	100	9,768	88.1 (87.0–89.1)	10,925	909	7.5 (6.7–8.3)	8.6 (6.9–10.3)	9,975	90.0 (88.9–90.9)	NA	NA	NA
All 18–99-yr-olds	10,250	100	9,247	89.1 (88.0–90.1)	10,245	866	7.8 (7.0–8.6)	8.9 (7.2–10.7)	9,439	91.0 (90.0–92.0)	NA		NA
**Sex**													
Women	5,897	51.1	5,272	89.2 (87.9–90.3)	5,892	500	7.2 (6.3–8.2)	8.4 (6.5–10.5)	5,377	90.6 (89.3–91.7)	Ref.	0.51	90.2 (89.0–91.4)
Men	5,035	48.9	4,496	87.0 (85.4–88.4)	5,033	409	7.8 (6.7–9.0)	8.9 (6.8–11.2)	4,598	89.3 (87.8–90.7)	0.94 (0.77–1.14)		89.7 (88.3–91.0)
**Age group**													
14–17 yrs	682	8.2	521	77.1 (71.9–81.6)	680	43	4.3 (2.9–6.5)	5.1 (2.6–9.8)	536	78.1 (72.9–82.5)	0.51 (0.36–0.72)	< 0.0001	77.3 (72.2–81.7)
18–34 yrs	1,880	22.1	1,616	84.6 (81.8–87.0)	1,879	181	8.1 (6.6–9.8)	10.5 (7.1–15.3)	1,671	87.5 (84.8–89.7)	Ref.		86.8 (84.1–89.1)
35–49 yrs	2,412	20.6	2,068	84.2 (81.5–86.6)	2,411	239	11.4 (9.3–13.9)	12.8 (9.7–16.3)	2,136	87.7 (85.2–89.8)	1.11 (0.82–1.50)		88.0 (85.5–90.0)
50–64 yrs	3,442	25.6	3,140	90.6 (88.9–92.0)	3,441	275	6.5 (5.3–7.8)	6.6 (4.9–8.7)	3,188	91.9 (90.3–93.3)	1.78 (1.33–2.37)		92.0 (90.5–93.3)
65–99 yrs	2,516	23.5	2,423	95.9 (94.4–96.9)	2,514	171	5.7 (4.7–7.0)	6.4 (4.6–8.6)	2,444	96.4 (95.0–97.4)	4.23 (2.78–6.44)		96.5 (95.1–97.5)
**Socioeconomic deprivation** ^f^													
Low	2,654	26.0	2,431	90.6 (88.6–92.3)	2,652	203	7.2 (5.7–9.2)	8.4 (5.7–11.8)	2,477	92.1 (90.1–93.7)	Ref.	0.11	92.1 (89.9–93.8)
Medium	6,645	59.9	5,902	87.4 (85.9–88.8)	6,640	563	7.5 (6.6–8.5)	8.3 (6.5–10.1)	6,035	89.4 (88.0–90.7)	0.72 (0.52–1.00)		89.4 (88.0–90.7)
High	1,535	14.1	1,346	86.5 (83.2–89.2)	1,535	133	7.8 (6.0–10.1)	9.7 (6.6–14.1)	1,371	88.5 (85.4–91.1)	0.64 (0.40–1.01)		88.3 (84.8–91.1)
**Region**													
Northern	1,987	17.8	1,828	89.6 (86.8–91.8)	1,985	107	5.0 (3.8–6.5)	5.5 (3.3–8.2)	1,849	90.5 (87.9–92.7)	0.75 (0.52–1.07)	< 0.0001	90.6 (88.0–92.7)
Western	3,445	34.7	3,166	90.4 (88.5–91.9)	3,442	279	7.8 (6.5–9.4)	8.8 (6.6–11.5)	3,231	92.7 (91.1–94.0)	Ref.		92.7 (91.1–94.1)
Southern	3,090	29.6	2,773	89.0 (86.9–90.8)	3,089	246	6.7 (5.3–8.3)	7.6 (5.2–10.8)	2,821	90.1 (88.0–91.8)	0.64 (0.45–0.91)		89.2 (86.6–91.4)
Eastern	2,410	18.0	2,001	80.8 (77.6–83.6)	2,409	277	10.7 (8.9–12.9)	12.9 (9.9–16.5)	2,074	84.0 (81.0–86.6)	0.44 (0.32–0.60)		85.4 (82.5–87.8)

CI: confidence interval; NA: not applicable; OR: odds ratio; Ref.: reference; RKI-SOEP-2: second Corona Monitoring Nationwide study based on a population sample embedded in a dynamic cohort (the Socio-Economic Panel; SOEP); SARS-CoV-2: severe acute respiratory syndrome coronavirus 2.

^a^ The RKI-SOEP-2 study collected data between 13 November 2021 and 6 March 2022 but these were predominantly obtained between November 2021 and January 2022.

^b^ Percentage with hybrid immunity, without correction of antibody results for test characteristics and antibody waning.

^c^ Prevalence of hybrid immunity, with correction of anti-N antibody results in vaccinated participants for anti-N specificity (= 0.993), sensitivity and antibody waning as described in Supplement 6.3.

^d^ Prevalence of basic immunisation not corrected for test characteristics and antibody waning, since correction leads to similar results (data not shown).

^e^ The model-adjusted ORs and prevalences are mutually adjusted for the variables in the table and month of study participation (as categorical variable). They are based on the 10,834 participants with information to determine basic immunisation and for whom information on all variables and study participation month was also available (see footnote f). Exponentiated Wald CIs and p-value for Wald test of each variable in survey logistic regression.

^f^ Numbers do not add up to a total of 10,932 due to 98 missing values in the variable for socioeconomic deprivation (available-case analysis).

All percentages are population weighted. All analyses for 14–99 years unless otherwise specified.

In multivariable logistic regression models, combined seroprevalence was associated with age group, socioeconomic deprivation and region (see Supplement 11 for a map and for stratification by federal state). Infection status and basic immunisation were associated with age group and region. Model-adjusted prevalence estimates differed only slightly from unadjusted estimates. There was no statistically significant association with sex, although men seemed to be at a somewhat higher infection risk than women (OR: 1.19; 95% CI: 0.87–1.66). Compared with adults aged 18–34 years, adolescents (14–17 years) were both significantly less likely to show combined seropositivity (OR: 0.34; 95% CI: 0.21–0.53) and to have a basic immunisation (OR: 0.51; 95% CI: 0.36–0.72). The odds of showing combined seropositivity were also reduced in areas with high socioeconomic deprivation (OR: 0.46; 95% CI: 0.21–0.96 for high vs low) and in the eastern region (OR: 0.29; 95% CI: 0.18–0.47, vs western region). The older age groups were both more likely to have a basic immunisation (OR: 1.78; 95% CI: 1.33–2.37 and OR: 4.23; 95% CI: 2.78–6.44 for 50–64 and 65–99 years, respectively) and less likely to have had an infection (OR: 0.46; 95% CI: 0.25–0.75 and OR: 0.48; 95% CI: 0.25–0.83, respectively). Participants from the eastern and southern regions were less likely to have a basic immunisation (OR: 0.44; 95% CI: 0.32–0.60 and OR: 0.64; 95% CI: 0.45–0.91, respectively) than participants from the western region. Individuals from the east were also more likely to have had an infection (OR: 1.90; 95% CI: 1.21–3.15).

## Discussion

Our study shows that in autumn/winter 2021/2022, when the Omicron wave was beginning, 19 of 20 persons aged ≥ 14 years were seropositive for anti-SARS-CoV-2 antibodies in Germany. The remaining 5% of the population were still immunologically naïve. One in nine individuals had been infected with SARS-CoV-2, and nine in 10 had a basic immunisation, primarily from vaccination and to a much lesser extent (9%) from the combination of vaccination and infection (hybrid immunity). Only one in three infections had not been notified during the first 2 pandemic years combined.

The added value of our study derives from multiple aspects: (i) it is based on a representative nationwide sample, thus generalisable to the population, (ii) the information collected is very detailed, (iii) seroprevalence and the prevalence of hybrid immunity was corrected for antibody waning, (iv) we provide an estimate of under-reporting, (v) the study includes adolescents aged 14–17 years, who showed prevalence patterns different from adults, and (vi) it was possible to assess hybrid immunity at the individual level.

Our results are consistent with other nationwide studies in Germany. From September 2021 to April 2022, the SeBluCo study [20] in adult blood donors (aged ≥ 18 years) showed an increase in combined seroprevalence from 89.4% to 100%, consistent with our estimate of 95.5% for adults in between those time points. In summer 2022, the GUIDE [21] study in the general adult population found a seroprevalence of 95.7% for anti-S antibodies and 44.4% for anti-N antibodies (both uncorrected), which indicates a further consistent rise of anti-S antibody prevalence and reflects the strong increase in infections during the Omicron wave. A modelling study [22] of notified infections and vaccinations yielded a pre-Omicron proportion of immunologically naïve of 10.8% (in 18–59-year-olds) and 4.8% (in ≥ 60-year-olds), which is higher than in our data (5.5% and 2.2% for 18–59 and ≥ 60 years age groups, respectively). The discrepancy may be due to inaccuracies in notified vaccinations and to idealised assumptions in the modelling study, but it also indicates a possible overrepresentation of vaccinated individuals in our study, as discussed in Supplement 7.1. Our estimates for combined seroprevalence are similar to other nationwide European studies, among them Finland (90–91%, November to December 2021, in 18–70-year-olds), Portugal (92.2% and 94.1%, September to November 2021, in people aged ≥ 10 and ≥ 20 years, respectively), Estonia (89.9%, November 2021; 93.2%, January 2022, in ≥ 18-year-olds), Belgium (91.3%, January to February 2022, in ≥ 18-year-olds) and Scotland (91.5%, December 2021 to January 2022, in ≥ 0-year-olds; Supplement 10).

We estimate the extent of under-reporting in mandatory COVID-19 notification data over the first 2 pandemic years as 1.55 (95% CI: 1.3–1.8) for the population aged ≥ 14 years (1.62 for adults aged ≥ 18 years), which is lower than the one observed in adults after the first pandemic year (1.82; 95% CI: 1.3–2.5) [6]. This decrease over time is consistent with other studies [1,20] and can be explained by intensified diagnostics and improved access to SARS-CoV-2 testing over time. As in the first wave of the study, we found suggestive evidence of higher under-reporting in areas with higher infection rates (here: the eastern region). The SeBluCo study [20] also found higher under-reporting in the eastern region, but confined to the beginning of 2021 and accompanied by higher under-reporting also in the southern region. In the first wave of our study, there had been suggestive evidence of higher under-reporting in areas with high socioeconomic deprivation, which was not replicated here, indicating that regional inequalities in access to testing may have been reduced over time.

Consistent with previous findings [23-25], combined seroprevalence differed by age, region and socioeconomic deprivation, while the prevalence of infection and basic immunisation differed by age and region. The still relatively low infection rate recorded overall is most probably due to the fact that the majority of our data were collected just before the more transmissible Omicron variant became dominant [26]: the corrected infection-induced seroprevalence seemed to increase with time, being 10.3% (95% CI: 7.4%–13.2%) for individuals participating in November/December 2021 and 15.2% (95% CI: 11.0%–19.4%) for participants in January to beginning of March 2022, with an apparent stronger increase in the younger age groups (< 50 years; data not shown and subject to uncertainties, since early and late respondents may differ). The finding of higher infection rates, but lower basic immunisation rates, among younger age groups confirms similar findings in notification data from the Delta wave [27]. We observed the same phenomenon in the eastern region, as the GUIDE study [21], again supporting national surveillance data. Importantly, our data highlight how the higher prevalence of infection and of hybrid immunity detected in the eastern region is not sufficient to counterbalance the low prevalence of two vaccine doses when it comes to achieving a high level of basic immunisation. This corresponds to a modelling study demonstrating that the unvaccinated were the primary driver of the rise in new infections in the autumn of 2021 [28].

One of the major strengths of our study is the use of a nationwide, population-based sample covering all districts in Germany, drawn from a long-running dynamic cohort that allows for sophisticated weighting and thus higher generalisability. Although some selection bias may be present, in general our study population shows good comparability with national surveillance data (see Supplement 7). Another strength of our study is the high quality of the data collected. Participants were asked to provide detailed information on their infection and vaccination status, which enhances validity, and which allowed us to verify the time since last exposure as well as the time intervals between immunological events. By combining information on self-reported infections and vaccinations with antibody testing and correcting for sensitivity as estimated from the study population, we minimised the effects of antibody waning, which is particularly an issue for anti-N antibodies. Furthermore, our study estimates the prevalence of hybrid immunity (8.6% when the Omicron wave was beginning), which provides more robust immune response and increased protection from infection and severe disease compared with either vaccination or infection alone [2-5,29]. Finally, information collected in this population-based sample can be used for future analyses (e.g. on Long COVID).

Our findings must be interpreted considering potential sources of bias including the underrepresentation of specific settings such as long-term care facilities, which had increased infection rates during the first two pandemic waves and were highly prioritised during the vaccination campaign [30]. The selective participation of more health-conscious individuals may have led to some overestimation of vaccination coverage (see Supplement 7.1). At the same time, the proportion immunologically naïve, the proportion of individuals with past infection and the under-reporting factor will be somewhat underestimated. Since no characteristics directly related to the pandemic such as attitudes towards anti-pandemic measures could be included in the weighting process, this selective participation could not be entirely corrected for. However, even if we assume that 20% of the population had not been represented by the study, with an anti-N seroprevalence three times as high as in the study, the under-reporting factor would only become 2.17 instead of the observed 1.55. Some participation bias was found in the younger age groups, where individuals with known infections were underrepresented in the age groups 14–17 and 18–34 years, and overrepresented in the age group 35–49 years (see Supplement 7.2). Infection rates and under-reporting may therefore be underestimated in adolescents and young adults, and comparisons between the age groups 18–34 years and 35–49 years are uncertain, also because of different results for infection-induced seroprevalence than for infection status. Finally, the use of study-internal estimates of sensitivity, relying on diagnosed self-reported infections, depends on the assumption that sensitivity is equal for known and unknown infections, and that access to and use of PCR testing is homogeneously distributed in the strata. Ideally, sensitivity would be derived from a population that has regularly been screened for infections with high-sensitivity PCR tests; however, no such sample spanning 2 years of the pandemic with nationwide coverage is available. Sensitivity can be expected to be lower in asymptomatic and mild, thus undiagnosed infections [31,32] and may therefore be overestimated in the study, leading to some underestimation of infection-induced seroprevalence, hybrid immunity and under-reporting. The extent of this bias may vary between groups with different use of testing.

## Conclusion

By the turn of the 2021/22 winter, most of the German population aged ≥ 14 years had been vaccinated, infected, or both. Most had at least a basic immunisation which was primarily due to vaccination and to a much lower extent to hybrid immunity, reflecting the successful containment strategy. Our results show how large-scale vaccination, but not a high infection rate, was able to fill the immunity gap especially in older individuals (aged over 65 years) who are known to be at higher risk of severe COVID-19. However, a non-negligible proportion of adolescents aged 14–17 years (15%), young adults (6%, 18–34 years) and middle-aged adults (7%, 35–49 years) had not been exposed to SARS-CoV-2 antigens at the start of the Omicron wave. It will be of utmost importance to address socioeconomic, demographic and regional disparities when establishing prevention strategies in the future, including measures to enhance vaccination uptake.

## Supplementary Data

1196000 Supplementary Material
