# Supplementary material for: Nationwide population-based infection- and vaccine-induced SARS-CoV-2 antibody seroprevalence in Germany in autumn/winter 2021/2022
Source: Euro Surveill. 2025 Jan 9;30(1):2400037. doi: 10.2807/1560-7917.ES.2025.30.1.2400037 (PMC11719803; doi:10.2807/1560-7917.ES.2025.30.1.2400037)
Supplement: Supplementary Material [file 2400037_SupplementaryMaterial.pdf]

This supplementary material is hosted by Eurosurveillance as supporting information alongside the article "Nationwide population-based infection- and vaccine-induced SARS-CoV-2 seroprevalence in Germany in autumn/winter 2021/2022", on behalf of the authors, who remain responsible for the accuracy and appropriateness of the content. The same standards for ethics, copyright, attributions and permissions as for the article apply. Supplements are not edited by Eurosurveillance and the journal is not responsible for the maintenance of any links or email addresses provided therein.

Mercuri E, Schmid L, Poethko-Müller C, Schlaud M, Kußmaul C, Ordonez-Cruickshank A, Haller S, Rexroth U, Hamouda O, Schaade L, Wieler LH, Gößwald A, Schaffrath Rosario A:

Nationwide population-based infection- and vaccine-induced SARS-CoV-2 antibody seroprevalence in Germany in autumn/winter 2021/2022 – Supplementary information

Content

1. Background information: Vaccination coverage in Germany by age group ... 2
2. Laboratory measurements and internal validation study ... 3
  2.1. Laboratory methods and quality assessment ... 3
  2.2. Internal validation study: Application of the Euroimmun Anti-SARS-CoV-2-NCP (IgG) and Anti-SARS-CoV-2-QuantiVac-ELISA (IgG) antibody tests to dried blood spots ... 3
3. Definition of outcome variables ... 6
4. Estimation of the underreporting factor ... 9
5. Stratum-specific sensitivity estimates ... 9
  5.1. Combined seroprevalence ... 9
  5.2. Infection-induced seroprevalence ... 10
6. Correction for test characteristics and antibody waning ... 11
  6.1. Stratified analyses of seroprevalence ... 11
  6.2. Logistic regression with correction for test characteristics and antibody waning ... 11
  6.3. Correction for test characteristics and antibody waning in composite variables (e.g. hybrid immunity) ... 13
7. Comparison with national surveillance data ... 14
  7.1. Comparison with national vaccination monitoring ... 14
  7.2. Comparison with mandatory COVID-19 case notification ... 18
8. Stratified analysis of the components of infection status ... 20
9. Flowchart of the study population ... 21
10. Seroprevalence studies in other European countries ... 21
11. Results stratified by region and federal state ... 22

1. Background information: Vaccination coverage in Germany by age group

As a background information, the following figure provides an overview of the vaccination coverage in Germany over time and in different age groups, based on the national COVID-19 Electronic Vaccination Coverage Monitoring database (Digitales Impfquotenmonitoring, DIM) [1,2,5]. The vaccination campaign in Germany started on 27.12.2020, with older age groups (starting with 80+ years) and those at higher risk of severe disease or exposure (e.g. some medical professions, people living or working in long-term care facilities) being vaccinated first [3,4]. The inclusion of adolescents aged 12-17 years in the vaccination campaign was first approved on 28.05.2021, but recommended first only for adolescents with risk factors. Starting from 24.08.2021, vaccination was recommended for all adolescents in this age group [3]. In children aged 5-11 years, vaccination was generally recommended in December 2021. Basic immunisation started to rise in children aged 5-11 years (not included in our study) in mid-December 2021 [2].

There is practically no increase in the prevalence of basic immunisation in the age group 60+ years within the study period (November 2021 to beginning of March 2022), but there is still an increase of about five percentage points in the age group 18-59 years, and a steeper increase among adolescents aged 12-17 years.

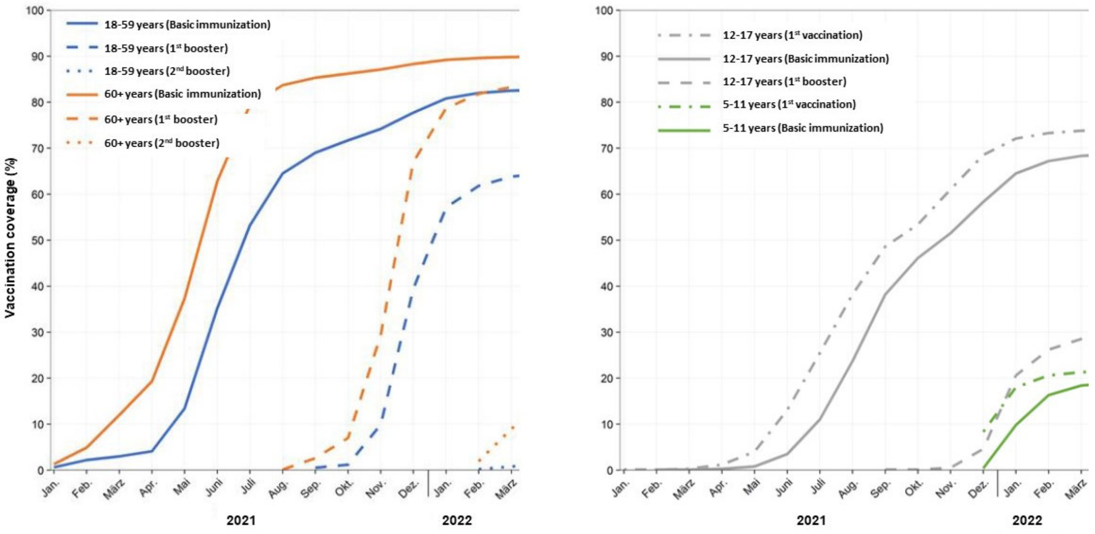

Supplementary Figure S1. Germany, January 2021 - March 2022. Source: COVID-19 Electronic Vaccination Coverage Monitoring database (Digitales Impfquotenmonitoring, DIM) [1], as of 31.01.2023. **Left panel:** Vaccination coverage, basic immunisation and booster vaccinations in adults (18-59 years, 60+ years). **Right panel**: Vaccination coverage, basic immunisation and booster vaccinations in children and adolescents (5-11 years, 12-17 years). Modified from Figures 3 and 4 in the monthly report on vaccination coverage [2]. Note that in the surveillance data, there is less information available for defining basic immunisation than in the study, the major difference being the assessment of vaccination with Jcovden (previously COVID-19 Vaccine Janssen) changed over time and that no antibody results are available in the surveillance data. Moreover, only infections that occurred before vaccination (not afterwards) can be counted in the DIM (see Supplement 7.1 for more details).

References

[1] Robert Koch-Institut. COVID-19-Impfungen in Deutschland. Zenodo. 2024. https://zenodo.org/records/12621034, accessed 04.07.2024.

[2] Robert Koch-Institut. Monitoring des COVID-19-Impfgeschehens in Deutschland. Monthly report, 02.02.2023. https://www.rki.de/DE/Content/Infekt/Impfen/ImpfungenAZ/COVID-19/Monatsberichte/2023-02-02.pdf?__blob=publicationFile, accessed 04.07.2024.

[3] Perumal N, Steffen A, Ullrich A, Siedler A. Impact of COVID-19 immunisation on COVID-19 incidence, hospitalisations, and deaths by age group in Germany from December 2020 to October 2021. Vaccine. 2022;40(21):2910-4.

[4] German National Standing Committee on Vaccination (STIKO). Stufenplan der STIKO zur Priorisierung der COVID-19-Impfung, February 2021. https://www.rki.de/DE/Content/Infekt/Impfen/ImpfungenAZ/COVID-19/Stufenplan.pdf?__blob=publicationFile, accessed 12.07.2024.

[5] Perumal N, Schönfeld V, Wichmann O. Application of the screening method for estimating COVID-19 vaccine effectiveness using routine surveillance data: Germany's experience during the COVID-19 pandemic, July 2021 to March 2023. Euro Surveill. 2024; 29: 2300329.

2. Laboratory measurements and internal validation study

2.1. Laboratory methods and quality assessment

Participants sent dried blood samples (DBS) by mail to the Epidemiological Central Laboratory of the Robert Koch Institute (RKI). Standardized punches of DBS (DBS Puncher, PerkinElmer, Waltham MA, USA) were then extracted according to the manufacturer's protocol (Euroimmun AG, Lübeck, Germany) and tested for SARS-CoV-2 anti-S1 (S1 domain of the spike protein) and anti-N (nucleocapsid protein, NCP) IgG antibodies. The Euroimmun ELISAs (enzyme-linked immunosorbent assays) anti-SARS-CoV-2-QuantiVac (QuantiVac) and anti-SARS-CoV-2-NCP (NCP) were used, respectively, for the quantitative detection of anti-S and for the semi-quantitative detection of anti-N antibodies. For defining seropositivity for anti-N antibodies, the ratio cutoff provided by the manufacturer for serum samples was adapted from 1.1 to 0.95 to account for the use of dried blood spots (see Supplement 2.2). All analyses were performed on a EUROLabWorkstation ELISA (Euroimmun AG, Lübeck, Germany), testing three quality control specimens (two provided by the manufacturer and one DBS control) on each 96-well plate. The Epidemiological Central Laboratory at the RKI is accredited according to DIN EN ISO 17025 and DIN EN ISO 15189 (Deutsche Akkreditierungsstelle, Frankfurt/Main, Germany). Internal quality assessments were conducted for both assays. For the quality control specimens used, intra-assay coefficients of variability (CV) ranged from 4.0% to 8.2% for the QuantiVac and 6.3% to 6.4% for the NCP. Inter-assay CVs ranged from 2.9% to 4.8% for the QuantiVac and 4.9% to 9.7% for the NCP. Relative root mean squared error (RRMSE) was used as a measure of accuracy and ranged from 4.4% to 18.5% for the QuantiVac and from 8.0% to 10.7% for the NCP. The laboratory participated in external quality assessments (EQAs) on the detection of SARS-CoV-2 IgG antibodies, offered by INSTAND interlaboratory comparison program (INSTAND, Düsseldorf, Germany) and passed all round robin tests on SARS-CoV-2 IgG antibodies.

2.2. Internal validation study: Application of the Euroimmun Anti-SARS-CoV-2-NCP (IgG) and Anti-SARS-CoV-2-QuantiVac-ELISA (IgG) antibody tests to dried blood spots

A previous validation study during the first wave of the RKI-SOEP-study revealed an adapted cutoff of 0.94 for classifying semiquantitative values of the Euroimmun Anti-SARS-CoV-2-S1-IgG ELISA antibody test in dried blood spot (DBS) samples [1]. For the second wave of the study, different test assays were used. S-antibodies were analysed quantitatively (as opposed to the semiquantitative test in the first wave) using the Anti-SARS-CoV-2-QuantiVac-ELISA (IgG) by Euroimmun. N-antibodies, which could not yet be tested for in the first wave, were analysed semiquantitatively with the Anti-SARS-CoV-2-NCP (IgG) (also by Euroimmun). These tests are commonly used in the analysis of serum samples and are CE-certified for use on DBS. However, we conducted an additional validation study comparing serum with DBS results in May and June 2022 to potentially optimize the cutoff for DBS. A total of 244 employees of the RKI volunteered to take part in the study after an institute-wide call was published via email. Blood specimens were taken after participants' informed consent. The samples for all 244 participants could be evaluated. Results were reported back to participants anonymously and no additional information, e.g. on their age, sex, vaccination or infection history, was collected.

Study execution and laboratory methods

From each participant, the study team collected both a venous blood sample, which was processed into serum, and a capillary blood sample, which was processed into DBS. Both samples were then tested for IgG antibodies using the Anti-SARS-CoV-2-QuantiVac-ELISA (IgG) and Anti-SARS-CoV-2-NCP (IgG) (both by Euroimmun AG, Lübeck, Germany). The results of the S-antibody test were quantitative, expressed in binding antibody units (BAU/mL) and classified for serum samples according to the manufacturer's specifications (positive: ≥ 35.2 BAU/mL, indeterminate ≥ 25.6 to < 35.2 BAU/mL, negative: < 25.6 BAU/mL). The results of the N-antibody test were semiquantitative ratio values which were classified for serum samples using the manufacturer-supplied cutoffs (positive: ratio ≥ 1.1; indeterminate: 0.8 ≤ ratio < 1.1, negative: ratio < 0.8). The quantitative assay was

rerun with diluted samples for values above the upper detection limit; 27 samples remained above the upper detection limit even after dilution. After the analysis, all samples were discarded.

Statistical analysis

The aim of the analyses was to assess the test characteristics of the IgG test assays based on DBS compared to serum samples and, if appropriate, to derive an adapted cutoff and, in case of the quantitative S-antibody test, a potential correction formula for DBS results so that the seroprevalence and quantitative measures based on DBS are comparable to those based on serum samples. Results of the serum measurement were regarded as the gold standard for this analysis.

For the S-antibody test, a linear regression model was run for the log-transformed values in order to determine whether a correction formula was needed to predict quantitative serum values from DBS values. Values above the upper detection limit were set to the value of the upper detection limit. For sensitivity analysis, a model without the observations above the detection limit was run. Bland-Altman-Plots [2] were used to check for agreement visually. For this plot, the difference between the (log) serum value and the (log) DBS value is plotted against the mean of the two (log) values.

For both the quantitative S-antibody and the semiquantitative N-antibody test, the categorised values were examined for agreement and equal marginal frequencies between serum and DBS classifications using McNemar's test [3]. The categorisation used was 'positive' versus 'non-positive' (negative or indeterminate) using the manufacturer-supplied cutoffs. The categorisation for S-antibodies showed perfect agreement between serum and DBS results in our sample (see Supplementary Table S1), so further analysis of S-antibody misclassification was not necessary.

**Result of serum sample**	**Result of DBS**		**Total**
	**Positive**	**Non-positive**	
**Positive**	241 (98.8 %)	0 (0 %)	241 (98.8 %)
**Non-Positive**	0 (0 %)	3 (1.2 %)	3 (1.2 %)
**Total**	241 (98.8 %)	3 (1.2 %)	244 (100 %)

Supplementary Table S1. Categorised IgG S-antibody measurement in serum vs. DBS using the manufacturer-supplied cutoff, unweighted absolute and cell percentages.

For the N-antibody results, an adapted cutoff for DBS values was determined using the discordant proportion ratio [4], the ratio of the cell percentage of false positives to the cell percentage of false negatives. The null hypothesis of McNemar's test can be expressed as a discordant proportion ratio of 1. In our application this would indicate that the DBS result is not systematically biased towards false positives or false negatives, compared to the serum sample. For this purpose, cutoffs in the range of 0.80-1.50 were used to classify the DBS values. For each cutoff, the proportion of misclassified DBS test results in comparison to serum results was determined and the ratio of false-positive to false-negative results was calculated. The cutoff that led to the discordant proportion ratio closest to 1 was chosen as the adapted cutoff.

In the analyses of the N-antibody results, weights were used to account for difference in seroprevalence (the proportion of positive N-antibody results) between the validation study and the main study. This procedure ensures that the results of the validation study are applicable to the main study, since it makes the marginal probabilities for positive and negative DBS test results identical to those observed in the main study. N-antibody positive observations were weighted with the ratio of the raw proportion of positive test results (5.2% in the main study to 17.6% in the validation study resulting in a weight <1) and negative ones with the respective proportions for negatives (94.8% in the main study, 82.4% in the validation study resulting in a weight >1). McNemar's test, however, was conducted on the unweighted data. As a further check, the weighted discordant proportion difference (difference of the cell percentage of false positives minus that of false negatives) before and after cutoff adaptation was tested against the null hypothesis of a difference equal to zero [7].

Confidence intervals for the proportion of misclassified DBS test results were calculated using the Wilson score method [5,6]. For weighted percentages, logit confidence intervals were calculated (with survey procedures).

Results

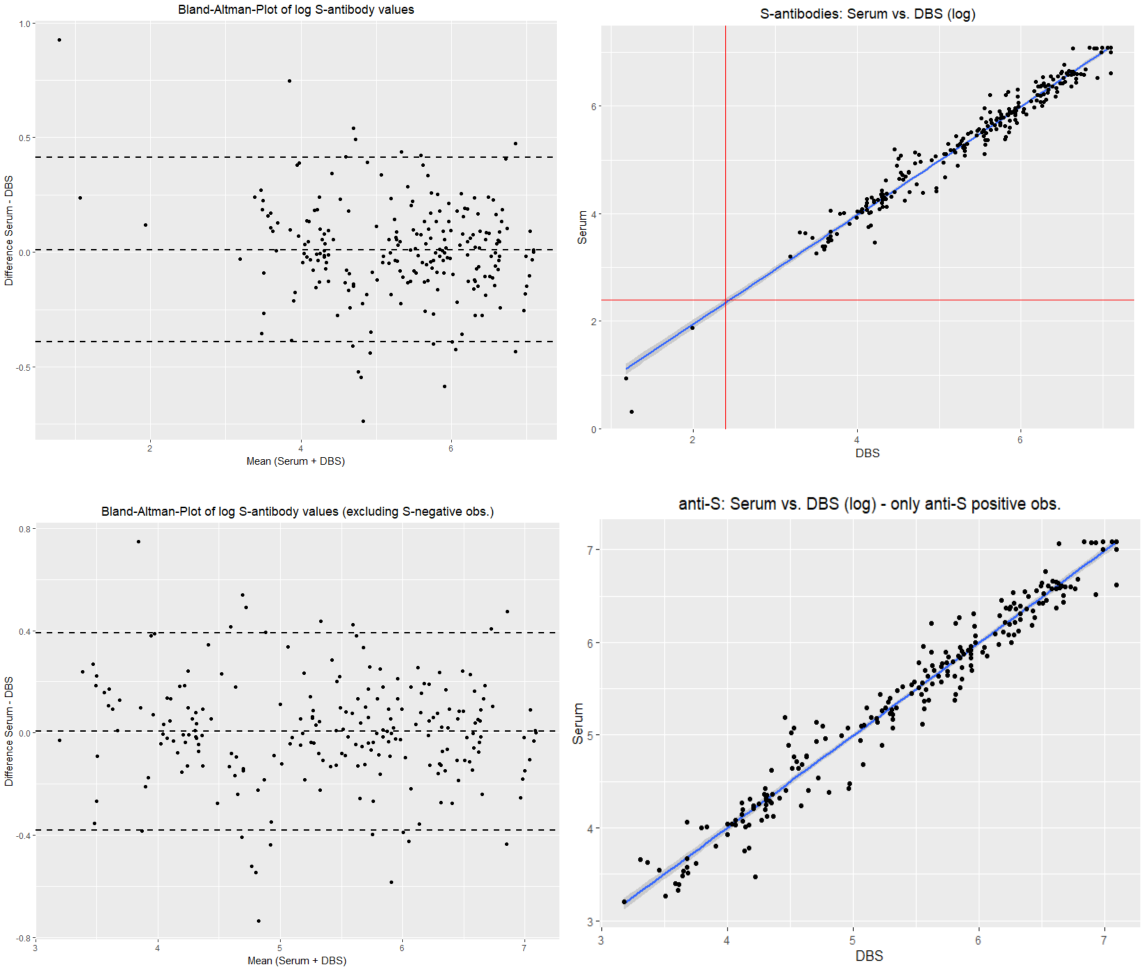

Supplementary Figure S2. Left panel: Bland-Altman plot of the difference between the quantitative serum log S1-IgG value and the corresponding DBS log value against the mean of the two log values. The dashed lines show the limits of agreement (± 1.96 standard deviations). Plots in the lower row exclude S-antibody negative observations. **Right panel**: Data points and regression line for the regression of quantitative serum log S1-IgG values on DBS log S1-IgG values. The red lines indicate the cutoff between positive and non-positive categorisation.

The linear regression models and Bland-Altman plots (see Supplementary Figure S2) indicated good agreement between serum and DBS values for the S-antibody test. For the S-antibody test, the explained variance (R^2) for the DBS values was 97.0%. The intercept (-0.078) had a standard error of 0.064 and was not significantly different from zero. The slope parameter (1.012) had a standard error of 0.011 and was not significantly different from 1. Since neither the intercept nor the slope was significantly different from 0 or 1, respectively, no correction formula was derived. Due to a slightly skewed Bland-Altman plot for the full sample, the analysis was repeated for the S-antibody positive observations only (lower row of Supplementary Figure S2), showing good agreement. Excluding the observations above the upper detection limit did not change this result, either.

For the categorised N-antibody test results, the proportion of misclassified DBS samples was 6.6% compared to the corresponding serum sample, applying the manufacturer-supplied cutoff of 1.1 to the DBS samples (16 of 244 dried blood samples were misclassified, 95% CI 4.1-10.4%) (see Supplementary Table S2). There were both false negative and false positive categorisations, whereby 12 (4.9%) positives in serum were false negatives (95% CI 2.8-8.4%) and 4 (1.6%) negatives in serum were false positives in DBS (95% CI 0.6-4.1%). McNemar's test without continuity correction provided support against the null hypothesis of equal marginal frequencies (p=0.0455), suggesting a statistically significant difference between the serum and DBS classifications. The estimated difference of discordant proportions was -5.2% (p=0.0015), supporting this interpretation. Adapting the cutoff for positivity for DBS values was therefore considered appropriate. The discordant proportion ratio closest to 1 was reached with a cutoff of 0.95. When re-applying McNemar's test to the marginal frequencies after cutoff adaptation, the null hypothesis could no longer be rejected on any meaningful significance level

(p=0.4227). The estimated difference of discordant proportions then was 0.5% (p=0.7478). The use of this cutoff led to an unweighted overall misclassification of 5.7% (14 of 244 samples misclassified, 95% CI 3.4-9.4%) and false positive and false negative misclassifications occurring with similar weighted frequency of 2.8% and 2.4% (5.2%, 12.7 of 244 samples misclassified, 95% CI 3.0-9.0%) (see Supplementary Table S2).

Implementation in the analysis of the main study

For IgG S-antibodies, the values of the quantitative Euroimmun QuantiVac ELISA were not found to differ significantly between serum and dried blood samples. For IgG N-antibodies, however, an adapted cutoff of 0.95 was obtained for classifying dried blood spot samples as N-antibody positive. This cutoff was therefore used to classify the semiquantitative values of the Euroimmun IgG N-antibody test in dried blood spot samples.

**DBS - Cutoff: 1.1**			
**Serum**<br>**DBS**	**Positive**	**Non-Positive**	**Total**
**Positive**	39 (4.8%)	4 (0.5%)	43 (5.2%)
**Non-Positive**	12 (5.7%)	189 (89.1%)	201 (94.8%)
**Total**	51 (10.4%)	193 (89.6%)	N=244
**DBS - Cutoff: 0.95**			
**Serum**<br>**DBS**	**Positive**	**Non-Positive**	**Total**
**Positive**	46 (8.1%)	9 (2.8%)	55 (10.9%)
**Non-Positive**	5 (2.4%)	184 (86.7%)	189 (89.1%)
**Total**	51 (10.4%)	193 (89.6%)	N = 244

Supplementary Table S2. Categorised IgG N-antibody measurement in serum vs. in dried blood spot using the manufacturer-supplied cutoff (1.1) and adapted cutoff (0.95), unweighted absolute numbers and weighted cell percentages

References

[1] Neuhauser H, Rosario AS, Butschalowsky H, et al. Nationally representative results on SARS-CoV-2 seroprevalence and testing in Germany at the end of 2020. *Scientific Reports.* 2022/11/14 2022;12(1):19492, Supplement: Application of the Euroimmun anti-SARS-CoV-19492-S19491-IgG ELISA antibody test to dried blood spots.

[2] Bland JM, Altman DG. Measuring agreement in method comparison studies. *Stat Methods Med Res.* Jun 1999;8(2):135-160.

[3] McNemar Q. Note on the sampling error of the difference between correlated proportions or percentages. *Psychometrika.* Jun 1947;12(2):153-157.

[4] SAS Institute Inc., The FREQ Procedure. SAS 9.4, https://documentation.sas.com/doc/en/pgmsascdc/9.4_3.3/statug/statug_freq_details76.htm SAS Institute, Cary NC. 2019. Accessed 23.02.2023.

[5] Newcombe RG. Two-sided confidence intervals for the single proportion: comparison of seven methods. *Stat Med.* Apr 30 1998;17(8):857-872.

[6] Wilson EB. Probable Inference, the Law of Succession, and Statistical Inference. *Journal of the American Statistical Association.* 1927/06/01 1927;22(158):209-212.

[7] SAS Institute Inc., PROC SURVEYFREQ. SAS 9.4, https://documentation.sas.com/doc/en/pgmsascdc/9.4_3.3/statug/statug_introsamp_sect006.htm, Cary NC. 2019. Accessed 14.03.2023.

3. Definition of outcome variables

Supplementary Table S3 illustrates which indicators were considered for defining each outcome variable. Combined seroprevalence and infection-induced seroprevalence were defined solely based on antibody test results. SARS-CoV-2 infection status and basic immunisation were defined based on the combination of information provided in the questionnaire and antibody test results.

Only participants with at least one available information on anti-S or anti-N IgG antibody test results were included in the analysis of combined seroprevalence. Any of self-reported positive PCR test, anti-N IgG antibody test result, or anti-S IgG antibody test result had to be available to be included in the analysis of SARS-CoV-2 infection status. The latter, the anti-S antibody test result, was only included for participants who reported never having been vaccinated. Sensitivity analyses requiring complete data yielded similar results. Not all dried blood spots sent in by the participants contained enough material to run analyses on both types of antibodies. In this case, only the anti-S test was carried out, leading to higher numbers of missing values for the anti-N test results.

	Anti-SARS-CoV-2 IgG antibodies		Self-reported vaccination	Self-reported infection	Time interval between events
	Anti-S	Anti-N			
**Combined seroprevalence**	+	+			
**Infection-induced seroprevalence**		+			
**Infection status**	(+)	+	(+)	+	
**Basic immunisation**		+	+	+	+

Supplementary Table S3. Indicators included in the definition of each outcome variable. Combined seroprevalence was defined as anti-N and/or anti-S seropositivity. Infection-induced seroprevalence was defined as anti-N seropositivity. Infection was defined based on self-reported positive PCR test or anti-N seropositivity. Participants with anti-S+ and anti-N- were also included in this category when they reported not having been vaccinated. This assumed that in the absence of reported vaccination, anti-S seropositivity was an indicator of previous infection despite having no anti-N seropositivity. Basic immunisation was defined as having a self-report of at least one vaccine dose and at least one additional exposure from vaccination (self-report) or infection (self-report or anti-N+). The time interval between immunologic events was evaluated to define basic immunisation (see text and Supplementary Figure S3).

Participants who reported a previous infection detected by PCR testing in the questionnaire were defined as participants with known infection. Participants with unknown infection were defined as those with no self-report of a positive PCR test, but positive for anti-N antibodies (independently of vaccination status) as well as those with no self-report of a positive PCR test and who reported never having been vaccinated, but were positive for anti-S antibodies (see Supplementary Figure S3).

To define basic immunisation, we counted the number (one, two, three, or four) of exposures to virus antigens as shown in Supplementary Figure S3. We considered individuals as having developed a basic immunisation if they had at least two exposures (from two or three doses of vaccine alone or from the combination of vaccination and infection). Based on the recommendations provided by the German National Standing Committee on Vaccination (STIKO) [1,2] at that time, we evaluated a vaccination (self-reported) and a subsequent infection (self-reported) as separate immunologically effective events only if a minimum time distance occurred between the two (Supplementary Figure S3). This distance was at least four weeks if the exposure from infection occurred after the first dose of vaccine, and at least three months if the infection occurred as third exposure after a second dose of vaccine. We assumed that the minimum time interval required between two subsequent doses of vaccine as well as between vaccination and prior infection was met as recommended by national immunisation guidelines at that time. A positive anti-N antibody test was considered a sign of an immunologically effective infection, even if the time requirements were not met. Anti-S antibody tests in the unvaccinated were not considered here, since a positive anti-S test would only indicate past infection and thus not fulfil the requirement of at least two antigen exposures. Only vaccines authorized by the European Medicines Agency (EMA) at the time of the study were considered (Comirnaty, Spikevax, Vaxzevria, JCOVDEN). Individuals vaccinated with JCOVDEN (previously COVID-19 Vaccine Janssen) were categorized as having a basic immunisation only if they had at least a second exposure from vaccination with an mRNA-based vaccine or infection. Of note, the definition of basic immunisation in the study differs somewhat from the definition in the national vaccination monitoring, since there is more information available in the study (see Supplement 7.1).

References

[1] Robert Koch Institute. Ständige Impfkommission: Beschluss der STIKO zur 20. Aktualisierung der COVID-19-Impfempfehlung. *Epid Bull.* 2022;21:3-19.

[2] Robert Koch Institute. Ständige Impfkommission: Beschluss der STIKO zur 21. Aktualisierung der COVID-19-Impfempfehlung und die dazugehörige wissenschaftliche Begründung. *Epid Bull.* 2022;33:3-19.

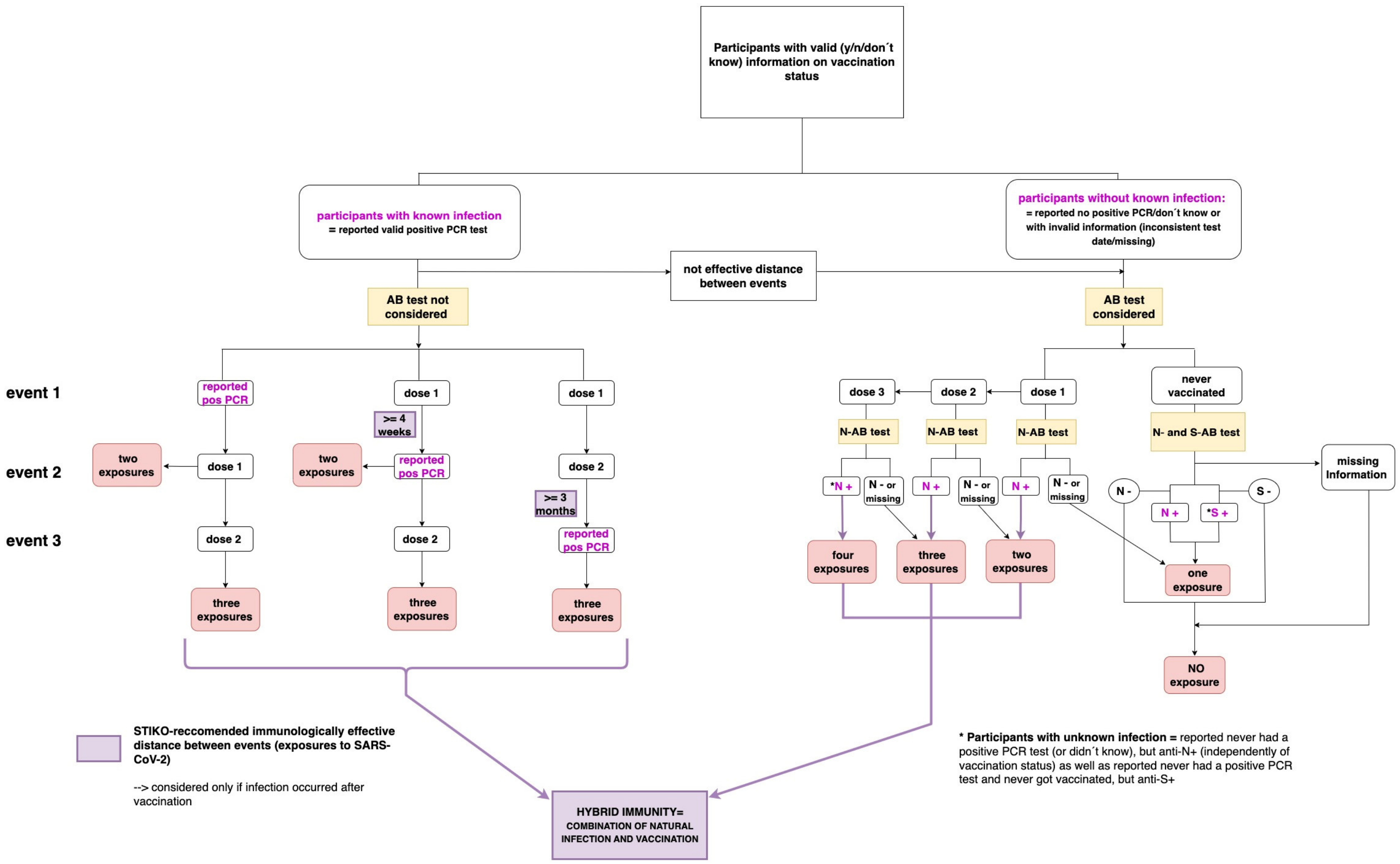

Supplementary Figure S3. Flowchart of the definition of basic immunisation. To define basic immunisation, we counted the number (one, two, three, or four) of exposures to the virus antigens, both from vaccination alone as well as from vaccination and infection (hybrid immunity). Individuals were categorized as having a basic immunisation if they had at least two exposures. See main text above for detailed explanations. Distance between events was evaluated only if the infection occurred after the vaccination. We assumed that the minimum time interval required between prior infection and vaccination (as well as between vaccine doses) was met as recommended by national immunisation guidelines at that time. The distance required for two subsequent immunologic events to be effective, was of at least four weeks if the infection occurred after the first dose of vaccine, and at least three months if the infection occurred after the second dose of vaccine.

4. Estimation of the underreporting factor

Underreporting of infections in COVID-19 case notifications was estimated by linking the study data to mandatory notification data [1]. To this end, we calculated the cumulative proportion of notified COVID-19 cases from the start of the pandemic until each day of the field period within strata defined by age group, sex and district. This prevalence of notified cases over the first two years of the pandemic was matched to participants by DBS sampling date (minus 14 days difference to symptom onset), age group, sex and district, and then averaged over all participants in the study (or over all participants in a stratum, for a stratified analysis) to yield an estimate of the cumulative proportion of non-fatal cases in COVID-19 case notification data. The underreporting factor was calculated as the ratio of infection-induced seroprevalence in the study (with correction for test characteristics and antibody waning) to the cumulative proportion of notified cases.

Deceased COVID-19 cases in the notification data were excluded from this calculation, since the study population cannot represent persons that have died from COVID-19, since only persons who are alive can take part in the study. The seroprevalence in the study is the seroprevalence among people who either have not been infected, or who have been infected, but have not died from COVID-19. Therefore, when comparing the seroprevalence in the study to the cumulative prevalence in the notification data, we should also restrict the notified cases to those who have not died from COVID-19.

Confidence intervals for the underreporting factor were obtained by dividing the CI limits for the corrected seroprevalence by the cumulative proportion of notified cases. *p*-values for pairwise comparisons of the categories of stratification variables were based on the difference of the log of the underreporting factors [2].

References

[1] Robert Koch-Institut. SARS-CoV-2 Infektionen in Deutschland. Zenodo. 2023.
[2] Neuhauser H, Rosario AS, Butschalowsky H, et al. Nationally representative results on SARS-CoV-2 seroprevalence and testing in Germany at the end of 2020. *Scientific reports.* 2022;12(1) Supplement 4, p. 10 ff.

5. Stratum-specific sensitivity estimates

Sensitivity was estimated internally from the study population instead of using the manufacturer-supplied value, in order to account for antibody waning as described in the main text. Here, we present the sensitivities estimated within categories of the stratification variables, for the stratified analyses of seroprevalence. Estimation of the sensitivity for the logistic regression analysis is described in Supplement 6.2. Estimation of the sensitivity for the analysis of hybrid immunity is described in Supplement 6.3.

5.1. Combined seroprevalence

The sensitivity for detecting combined seroprevalence was estimated by the proportion that was seropositive for anti-S or anti-N antibodies among study participants with a self-reported vaccination or a self-reported positive SARS-CoV-2 test at least 11 days pre-study (n = 9,260).

	Anti-S or anti-N seropositive					
	Sensitivity	(95%-CI)		N positive	*p*-value	N
**All (total 14-99 years)**	95.8%	(95.1 -	96.4)	8915		9260
**All (total 18-99 years)**	95.5%	(94.8 -	96.2)	8435		8771
**Women**	96.3%	(95.4 -	97.0)	4820	0.1152	4980
**Men**	95.3%	(94.1 -	96.2)	4095		4280
**14-17 years**	99.4%	(98.7 -	99.8)	480		489
**18-34 years**	97.4%	(95.5 -	98.5)	1580		1616
**35-49 years**	96.7%	(95.1 -	97.8)	2001		2051
**50-64 years**	95.8%	(94.5 -	96.9)	2870		2974
**65-99 years**	92.4%	(90.5 -	93.8)	1984	<.0001	2130

	Anti-S or anti-N seropositive					
	Sensitivity	(95%-CI)		N positive	*p*-value	N
**Low deprivation**	96.4%	(95.1 -	97.4)	2209		2286
**Medium deprivation**	96.0%	(95.1 -	96.8)	5456		5654
**High deprivation**	93.3%	(90.6 -	95.2)	1170	0.0107	1239
**Northern region**	94.5%	(91.9 -	96.3)	1637		1696
**Western region**	95.5%	(94.2 -	96.6)	2848		2960
**Southern region**	96.5%	(95.3 -	97.4)	2535		2627
**Eastern region**	96.4%	(95.3 -	97.3)	1895	0.1564	1977

Supplementary Table S4. Stratum-specific sensitivity for detecting combined seroprevalence, estimated from the study population. Population-weighted and taking clustering within households into account in the estimation of confidence intervals.

As a robustness check, we used additional stratification by the number of vaccinations when estimating sensitivity, but results were not altered materially (data not shown).

5.2. Infection-induced seroprevalence

The sensitivity for detecting infection-induced seroprevalence was estimated by the proportion that was seropositive for anti-N antibodies among participants with a self-reported positive SARS-CoV-2 test at least 11 days pre-study (n = 774).

	Anti-N seropositive					
	Sensitivity	(95%-CI)		N positive	*p*-value	N
**All (total 14-99 years)**	47.4%	(41.6 -	53.3)	358		774
**All (total 18-99 years)**	45.5%	(39.6 -	51.5)	322		710
**14-17 years**	65.2%	(45.4 -	80.9)	36		64
**18-34 years**	35.0%	(25.3 -	46.1)	61		160
**35-49 years**	47.3%	(36.9 -	57.9)	103		228
**50-64 years**	48.9%	(39.1 -	58.8)	113		231
**65-99 years**	55.6%	(41.0 -	69.2)	45	0.0390	91
**Women**	45.7%	(38.3 -	53.4)	191		426
**Men**	49.0%	(40.9 -	57.3)	167	0.5478	348
**Low deprivation**	51.3%	(38.3 -	64.2)	84		177
**Medium deprivation**	44.3%	(37.5 -	51.3)	227		495
**High deprivation**	59.6%	(45.8 -	72.0)	45	0.2190	95
**Northern region**	44.8%	(29.8 -	60.8)	40		75
**Western region**	46.1%	(36.0 -	56.5)	88		216
**Southern region**	48.3%	(37.2 -	59.5)	105		229
**Eastern region**	49.1%	(38.7 -	59.5)	125	0.9658	254

Supplementary Table S5. Stratum-specific sensitivity for detecting infection-induced seroprevalence, estimated from the study population. Population-weighted and taking clustering within households into account in the estimation of confidence intervals.

6. Correction for test characteristics and antibody waning

6.1. Stratified analyses of seroprevalence

In stratified analyses of combined and infection-induced (anti-N) seroprevalence, the correction for test characteristics and antibody waning was done by applying the usual Rogan-Gladen formula [1]:

$$prevalence_{corrected} = \frac{prevalence_{observed} + specificity - 1}{sensitivity + specificity - 1} \quad .$$

95% confidence intervals for the corrected seroprevalence were obtained by taking into account that sensitivity and specificity were estimated from data (the study data for sensitivity, external data [2] for specificity) and thus not fixed values, but subject to random variability. Thus, following the formula for *Var* ($\hat{p}_2$) in the appendix of Rogan and Gladen [1], the standard error used for these confidence intervals was calculated as

$$SE_{corrected} =$$

$$\sqrt{SE1^2 + SE2^2 \cdot (prevalence_{corrected})^2 + SE3^2 \cdot (1 - prevalence_{corrected})^2} / (sensitivity + specificity - 1)$$

where SE1² is the squared standard error of the estimated uncorrected seroprevalence, SE2² is the squared standard error of the estimated stratum-specific sensitivity, and SE3² is the squared standard error of the estimated specificity. For SE1 and SE2, we used the robust standard errors provided by the survey procedures. SE3² was calculated according to the standard formula $\frac{1}{N} \cdot specificity \cdot (1 - specificity)$ with N = 676. The 95% confidence intervals were then calculated as Wald intervals:

$$95\%\ CI = [prevalence_{corrected} \pm t_{0.975} \cdot SE_{corrected}] \quad ,$$

using the t-quantile $t_{0.975}$ based on the degrees of freedom determined by the survey procedure in the estimation of the uncorrected seroprevalence.

6.2. Logistic regression with correction for test characteristics and antibody waning

In logistic regression models of combined seroprevalence and infection-induced seroprevalence, we used the method of predictive value weighting[3].

The first step was to estimate the *sensitivity*. This was done similarly as described in Section 5.1 for the stratified analysis, only that here sensitivity was estimated via a logistic model, using the survey weights, with the same covariates as in the following analysis of seroprevalence (sex, age group, socio-economic deprivation, region and month of participation). As for the stratified analysis, the estimation of sensitivity was done in study participants with a self-reported vaccination or a self-reported infection at least 11 days pre-study (n = 9,260). Sensitivity was given by the probability of being seropositive, given the values of the covariates, as predicted by the logistic regression equation.

Second, an uncorrected logistic regression model was run, using the same model variables and the weights, in order to predict the *uncorrected seroprevalence* for each combination of the covariates. Thus, for each participant there was prediction of the uncorrected seroprevalence, based on the values of the covariates and the regression coefficients, and the estimated sensitivity from step 1, also dependent on the values of the covariates. These were then used to arrive at seroprevalence estimates corrected for test characteristics, based on the values of the covariates, by applying the usual correction for sensitivity and specificity [1] for each combination of the covariates, similar to stratified analysis (Supplement 6.1):

$$prevalence_{corrected} = \frac{uncorrected\ prevalence_{predicted\ by\ logistic\ model} + specificity - 1}{sensitivity_{predicted\ by\ logistic\ model} + specificity - 1} \quad .$$

Thus, there was a *corrected seroprevalence* for each combination of covariate values. However, the aim of logistic regression is to provide odds ratio estimates. Therefore, we applied the method of predictive value weighting [3].

Predictive value weighting is based on the idea that the observed antibody test results might be in error, i.e. that an observed positive result could be false-positive and should in fact be negative. The probability that an

observed positive result corresponds to a true positive result is called the positive predictive value (PPV). The probability that an observed positive result is erroneous and should in fact be negative is then given by 1 – PPV. In the same way, each observed negative test result is truly negative with a probability given by the negative predictive value (NPV), and it is in error and thus corresponding to a truly positive result with probability 1 – NPV.

So in the third step, the positive and negative predictive values were estimated for each combination of covariate values, according to standard formulae [4],

$$PPV = \frac{sensitivity \cdot corrected\ prevalence}{sensitivity \cdot corrected\ prevalence\ +\ (1 - specificity) \cdot (1 - corrected\ prevalence)}$$

$$NPV = \frac{specificity\ \cdot\ (1\ -\ corrected\ prevalence)}{(1 -\ sensitivity) \cdot\ corrected\ prevalence\ +\ specificity \cdot (1\ -\ corrected\ prevalence)}$$

using the model-based sensitivity (step 1) and the corrected model-based seroprevalence (step 2).

In the fourth step, all observations were duplicated to form pseudo-observations. The original observations were assigned a weight (the predictive value weight) given by PPV, if the observed test result was positive, since PPV is the probability that the observed result is correct (and NPV as predictive weight if the observed test result was negative). In the pseudo-observations, the observed "positive" was set to "negative" (which is true with probability 1 – PPV) and the predictive value weight was thus given by 1 – PPV. Similarly, in the pseudo-observations, observed negative values were set to "positive" and assigned a predictive weight of 1 – NPV. The predictive value weights sum up to 1 for the two observations (original and pseudo) created for each participant. See the examples in Supplementary Table S6.

ID	Type of observation	Observed antibody result	True antibody state	Observed sero-prevalence[a]	Estimated sensitivity[a]	Corrected sero-prevalence[a]	Predictive value weight [a,b]	Survey weight	Weight for final logistic model
**1**	Original	Positive	Positive	0.900	0.967	0.931	PPV = 0.9995	0.81	0.8096
**1**	Pseudo	Positive	Negative	0.900	0.967	0.931	1 – PPV = 0.0005	0.81	0.0004
**2**	Original	Negative	Negative	0.900	0.967	0.931	NPV = 0.69	1.33	0.9177
**2**	Pseudo	Negative	Positive	0.900	0.967	0.931	1 – NPV = 0.31	1.33	0.4123

Supplementary Table S6. Calculation of predictive value weights for two hypothetical participants from the same group (e.g. female, 35-49 yrs, district with medium deprivation, western region, participation in November).
PPV = positive predictive value, NPV = negative predictive value, specificity 0.994.
[a] Values are identical for all observations from the same group.
[b] The predictive value weight corresponds to the probability that, given the observed result, the true antibody result is given by the value in the column "True antibody state". The predictive value weights sum up to 1 for the two observations (original and pseudo) created for each participant (ID 1 and ID 2 in this example).

Finally, in step 5, the predictive value weights were multiplied with the survey weights and used in the logistic regression analysis of the combined dataset of original and pseudo-observations with the assumed true antibody state as target variable.

95% confidence intervals for the odds ratios and the adjusted prevalences derived from the model were estimated via a bootstrap procedure with 2000 replications, bootstrapping the whole process starting from the model-based estimation of sensitivity (step 1 above) until fitting the logistic model using the survey weights multiplied by the predictive value weights (step 5 above). Moreover, following [5], in each bootstrap replicate a new value for the specificity was simulated. Therefore, the confidence intervals not only reflect the random uncertainty resulting from the estimation of seroprevalence, but also the uncertainty stemming from estimating sensitivity and specificity as well as the uncertainty from estimating the predictive values. In detail, we took the following steps:

(a) We selected 2000 bootstrap samples from the original data set, sampling $m - 1$ clusters (households) from all households, with replacement, where m is the total number of households in the analysis population (m = 6476 in the logistic regression model for combined seroprevalence, m = 6340 in the model for infection-induced seroprevalence), using the SAS procedure PROC SURVEYSELECT (SAS 9.4, SAS Institute Inc., Cary, NC, USA). Due to unequal household sizes, the number of individuals included in each bootstrap sample varied slightly.

(b) As suggested by one of the reviewers and following the procedure established in a recent seroprevalence study in North Carolina, USA [5], for each of the 2000 bootstrap replicates, we simulated a value for specificity by a random draw from a binomial distribution with parameters n = sample size of the external population used for estimation of specificity, and p = the estimated value of the specificity, both as given in [2].

(c) For each replicate, we estimated the study-internal sensitivity based on a logistic regression model (step 1 above).

(d) For each replicate, we estimated the model-based positive and negative predictive values (steps 2 and 3 above).

(e) For each replicate, we generated pseudo-observations and calculated predictive value weights for the original as well as the pseudo-observations (step 4 above).

(f) For each replicate, we fitted the logistic regression, using the survey weights multiplied by the predictive value weights (step 5 above), and stored the resulting odds ratio estimates as well as the estimated model-adjusted prevalences.

(g) From the 2000 bootstrap estimates for each OR and each model-adjusted prevalence, 95% confidence intervals for the odds ratios and the model-adjusted prevalences were determined by the percentile method, using the respective 2.5% and 97.5% percentile as the lower and upper confidence limit.

We did not re-calculate the sample weights for each bootstrap replicate, as this was not feasible due to the complex modelling of contactability and participation probabilities underlying the sample weights. This might have led to confidence intervals that are somewhat conservative (i.e. have somewhat larger width).

6.3. Correction for test characteristics and antibody waning in composite variables (e.g. hybrid immunity)

For composite variables such as hybrid immunity, which combines information from the questionnaire with laboratory results, the usual correction formula (Supplement 6.1) cannot be applied, since this correction only affects the laboratory result.

In this section, we describe the correction for the variable “hybrid immunity”. We applied the method in a similar manner to the variable “infection status”. However, we do not show the results, since they were very similar to corrected infection-induced seroprevalence.

The variable “hybrid immunity” combines questionnaire information (which is assumed to be error-free) on vaccinations and past PCR-confirmed infections with the anti-N antibody test result. The antibody test result is only relevant in participants who report a vaccination, but no infection, since (1) unvaccinated persons cannot have hybrid immunity, since hybrid immunity is – by definition - present when both vaccination and infection have occurred, and (2) vaccinated participants with a self-reported infection are categorized as having hybrid immunity, regardless of their antibody test result (cf. Supplementary Figure S3).

Therefore, the correction for test characteristics and antibody waning was only done in the subgroup of participants who self-reported at least one vaccination, but no PCR-confirmed infection. Only for these participants, the method of predictive value weighting [3] was applied, as described in Supplement 6.2 for logistic regression models, the difference being that steps 1-3 were not applied in logistic regression models, but in stratified analyses using the same strata as in the main analysis. Of note, the estimation of stratum-specific anti-N sensitivity was done as described in Supplement 5.2, but restricted to participants with vaccination (n = 608; results not shown).

In the fourth step, pseudo-observations were formed only for the subgroup where the correction was applied, and variable definitions in the pseudo-observations were as follows: positive anti-N antibody results were set to "negative", negative results to "positive". Then, the assessment of hybrid immunity status was repeated in the same way as had been done for the original observations, only with the altered anti-N antibody result.

Finally, in step 5, the survey weights were multiplied with the predictive value weights for the participants in the subgroup where the correction was carried out, and remained unaltered for the remaining participants. The stratified analysis of hybrid immunity was then carried out on the combined dataset of the original and pseudo-observations for the subgroup with correction, and the usual original observations for the remaining participants.

95% confidence intervals were estimated via a bootstrap procedure with 2000 replications, as described for logistic regression (Supplement 6.2). The bootstrap samples were drawn from all participants (using the households as sampling units), and within each bootstrap sample, the correction was performed for the subgroup of participants who self-reported at least one vaccination, but no PCR-confirmed infection.

References

[1] Rogan WJ, Gladen B. Estimating prevalence from the results of a screening test. American J Epidem. 1978;107(1):71-6.

[2] Scheiblauer H, Nubling CM, Wolf T, et al. Antibody response to SARS-CoV-2 for more than one year - kinetics and persistence of detection are predominantly determined by avidity progression and test design. Journal of clinical virology: the official publication of the Pan American Society for Clinical Virology 2022; 146: 1-9.

[3] Lyles RH, Lin J. Sensitivity analysis for misclassification in logistic regression via likelihood methods and predictive value weighting. Stat Med. 2010;29(22):2297-309.

[4] Lewis FI, Torgerson PR. A tutorial in estimating the prevalence of disease in humans and animals in the absence of a gold standard diagnostic. Emerg Themes Epidemiol. 2012;9(1):9.

[5] Vias NP, Cassidy CA, Edwards JK, Xiong K, Beatty Parker C, Aiello AE, et al. Estimation of SARS-CoV-2 Seroprevalence in Central North Carolina: Accounting for Outcome Misclassification in Complex Sample Designs. Epidemiology. 2023;34(5):721-31.

7. Comparison with national surveillance data

7.1. Comparison with national vaccination monitoring

Supplementary Table S7 and Supplementary Figure S4 compare the population-weighted prevalence of basic immunisation in the study with the coverage of basic immunisation derived from the national COVID-19 Electronic Vaccination Coverage Monitoring database (Digitales Impfquotenmonitoring, DIM [1-4]). We used a special version of the DIM data that included vaccinations delivered until 31 December 2021 [2]. This data set was more complete and offered finer age granulation than the standard data set available on Github [1]. Data in the vaccination monitoring derive from vaccination centres, doctors and other places offering vaccination, capturing the aggregated number of daily vaccinations, grouped by type of vaccination (first vaccination vs. basic immunisation vs. booster), federal state (according to place of vaccination) and age group. In the standard data set, vaccination coverage is only available for broad age groups (18 to 59 years, 60 years and older). In the special data set [2], vaccination coverage is available in 10-year age groups (18 to 29 years, 30 to 39 years, …, 70 to 79 years, ≥ 80 years), additional information from health insurance claims data is included, and the assignment of vaccinations to the federal states is based on the place of living, not the place of vaccination. In the analyses presented here, federal state is not taken into account, however.

In order to match the DIM data used in the comparison [2], we restricted the study data to persons who participated in 2021, and we restricted both the study data and the DIM data to persons ≥ 18 years old (n = 7,061 in the study).

The definition of basic immunisation in this section of the Supplement differs from the definition used in the main part of the paper. It was adapted to the information available in the DIM. Therefore, only vaccinations and known infections are taken into account, not antibodies. As only infections before a vaccination can be considered in the DIM vaccination monitoring (because data in the reporting system are updated only once, when vaccination occurs), the study variable used in this section also only considers infections prior to a vaccination. Moreover, the definition of basic immunisation with the JCOVDEN vaccine (previously COVID-19 Vaccine Janssen) was adapted to the definition in use in the DIM during 2021, meaning that a single dose of JCOVDEN was considered sufficient for achieving basic immunisation. Basic immunisation was thus defined as having received at least two self-reported vaccinations, or at least one vaccination after a previous self-reported infection, or at least one self-reported dose of JCOVDEN vaccine.

Vaccination coverage in the DIM is calculated as the ratio of the reported number of vaccinations to official population numbers. When calculating basic immunisation rates for this section of the Supplement, some modifications were undertaken as compared to the analyses in [2]. The analyses in [2] do not contain information on vaccinations paid by private health insurances, which account for about 1% of all vaccinations [2]. Moreover, 0.6% of vaccinations were excluded because of missing information on the postcode of the place of living. We corrected for these exclusions by multiplying the reported number of vaccinations by $1/(0.99 \cdot 0.994)$, irrespective of age or other characteristics. For the denominator, we used the mean of the official population numbers for 31 December 2020 and 31 December 2021. According to preliminary results of the German census undertaken in May 2022, these numbers somewhat overestimate the population size (by 1.5 percentage points overall), so we applied agegroup-specific corrections according to preliminary numbers published by the Federal Statistical Office [5]. Without these corrections, the prevalence of basic immunisation would be somewhat underestimated by the DIM.

We present two comparisons of the DIM vaccination coverage with the study results. First, in Supplementary Table S7, each study participant is assigned the DIM rate of basic immunisation according to his or her 10-year age group and participation date (counting all basic immunisations reported until the calendar week when participating). Sex and federal state are not taken into account in this step. A weighted average of the assigned basic immunisation rate over each age group then represents the DIM vaccination coverage for this group, considering the different dates of study participation. This calculation is analogous to the estimation of under-reporting of infections (see Supplement 4). Second, in Supplementary Figure S4, a Kaplan-Meier-type survival analysis is shown. For this figure, the timing of achieving basic immunisation over the course of the year 2021 is compared between the DIM and the RKI-SOEP-2 study. For each study participant, the date when basic immunisation was achieved is shown (which can be weeks or months before study participation), and participants without basic immunisation enter the analysis as censored observations (censored at the date of participation).

As with every register, it is reasonable to assume a certain degree of incompleteness in the DIM data, especially since the DIM was a register that was newly established during the pandemic. As rough approximations, we present results assuming 97% and 95% completeness of the DIM. These assumptions are reasonable when comparing with the completeness of cancer registries: 13 of 16 cancer registries in Germany achieved a completeness of 95% or more for the entity ‘All cancers’ [6]. Also, the inclusion of health insurance claims data in the special analysis [2] of the DIM data increased the rate of basic immunisation by 1.3 percentage points (in the entire population, including children and adolescents up to 17 years of age).

Results

The first column of Supplementary Table S7 shows the prevalence of basic immunisation estimated by the RKI-SOEP-2 study, with the definition of basic immunisation adapted to the DIM. Among study participants, the observed weighted prevalence of basic immunisation was 92.9% (95% CI: 91.8% - 93.9%). This estimate is almost two percentage points higher than the estimate in the main part of the paper (Table 3). The reason for this higher estimate is the fact that in the 2021 definition of basic immunisation used here, one single JCOVDEN dose was considered sufficient for requiring basic immunisation. This effect was stronger in the younger age groups (data not shown).

To illustrate the uncertainties involved with the estimation of the prevalence of basic immunisation on a national basis, we then compare two versions of calculating this prevalence from the DIM data (Supplementary Table S7). The denominator in the first version is the population on 31 December 2020 (i.e. beginning of 2021), in the second version the population on 31 December 2021 (i.e. end of 2021), both corrected with the census-derived adjustment factors described above. While the overall prevalence of basic immunisation is practically the same in both versions (80.5% vs. 80.6%), in the age groups ≥ 50 years the choice of the reference population accounts for a difference of 1.0 to 2.7 percentage points in the estimated vaccination coverage. Therefore, we based our further calculations on the average of the population numbers on 31 December 2020 and 31 December 2021.

	RKI-SOEP-2 Study	Prevalence of basic immunisation according to national vaccination monitoring (DIM) [b]					Difference		
	Prevalence of basic immunisation [a]	Assumed completeness of the DIM					Assumed completeness of DIM		
		100%	100%	100%	97%	95%	100%	97%	95%
	% (95%-CI)	% per population [c] 31.12.2020	% per population [c] 31.12.2021	% per population average of 31.12.2020 and 31.12.2021 [c]					
**18-29 yrs**	**91.1%** (87.1 - 93.9)	72.1%	72.7%	**72.4%**	**74.7%**	**76.2%**	**18.7%**	**16.4%**	**14.8%**
**30-39 yrs**	**86.7%** (82.6 - 89.9)	72.3%	72.2%	**72.2%**	**74.4%**	**76.0%**	**14.5%**	**12.2%**	**10.6%**
**40-49 yrs**	**90.2%** (87.1 - 92.6)	75.5%	76.0%	**75.8%**	**78.1%**	**79.7%**	**14.4%**	**12.1%**	**10.5%**
**50-59 yrs**	**95.0%** (93.0 - 96.4)	81.7%	83.1%	**82.4%**	**85.0%**	**86.7%**	**12.6%**	**10.0%**	**8.2%**
**60-69 yrs**	**96.0%** (94.3 - 97.2)	87.0%	85.1%	**86.1%**	**88.7%**	**90.6%**	**9.9%**	**7.3%**	**5.4%**
**70-79 yrs**	**97.8%** (96.4 - 98.7)	91.1%	92.1%	**91.6%**	**94.5%**	**96.5%**	**6.2%**	**3.3%**	**1.3%**
**80+**	**96.7%** (92.5 - 98.5)	94.3%	91.6%	**93.0%**	**95.8%**	**97.8%**	**3.7%**	**0.8%**	**-1.2%**
**Total**	**92.9%** (91.8 - 93.9)	80.5%	80.6%	**80.6%**	**83.1%**	**84.8%**	**12.4%**	**9.9%**	**8.1%**

Supplementary Table S7. Comparison of prevalence of basic immunisation observed in the RKI-SOEP-2 study with the national COVID-19 Electronic Vaccination Coverage Monitoring database (Digitales Impfquotenmonitoring, DIM) [b] .

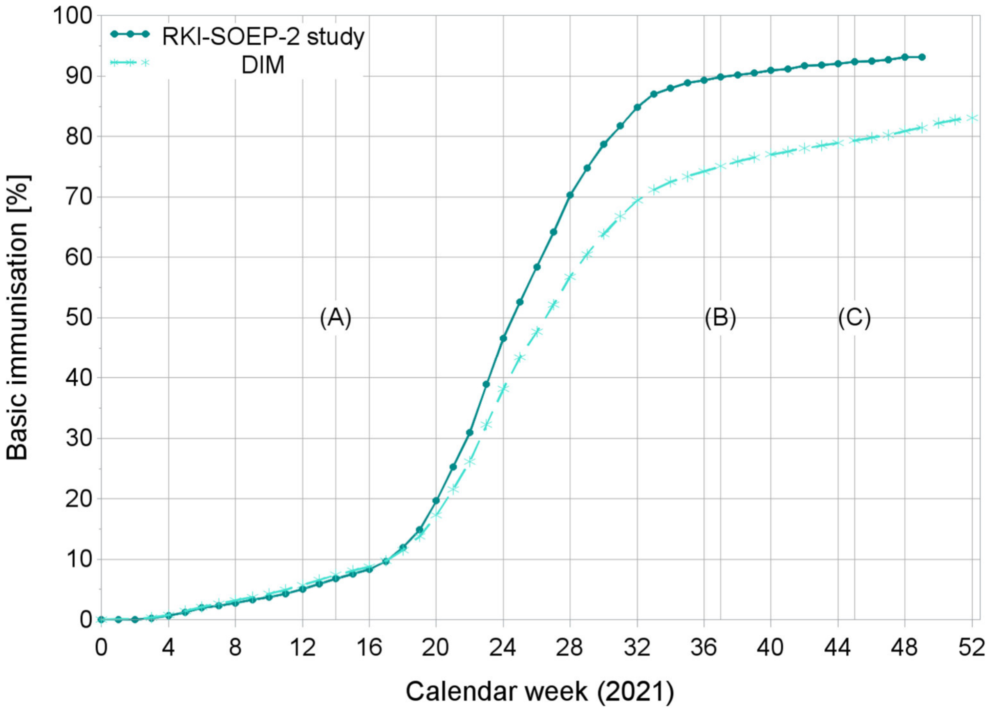

Supplementary Figure S4. Comparison of prevalence of basic immunisation [a] observed in the RKI-SOEP-2 study [d] with COVID-19 Electronic Vaccination Coverage Monitoring database (Digitales Impfquotenmonitoring, DIM), by calendar week. Starting from week 14 **(A)**, vaccinations were also offered by primary healthcare providers. In week 37 **(B)**, the first information regarding the study was sent to the members of the SOEP panel. Week 45 **(C)** marks the start of field work.

All percentages from the study data are population-weighted.
All analyses for persons 18-99 years, with study participation from 13 November to 31 December 2021.

[a] Study definition of basic immunisation adapted to the definition in use in the DIM during 2021, as described in the text.
[b] Vaccination coverage adjusted for sampling density (i.e. each study participant is assigned the age group-specific national vaccination coverage in the DIM corresponding to the calendar week of participation and these coverage rates are averaged over all participants).
[c] Population numbers corrected according to preliminary census results, as described in the text.
[d] Vaccination coverage in the DIM presented by calendar week. For the study data, the calendar week was determined based on the date of the vaccination dose that lead to basic immunisation (i.e. the date of the second vaccination; or the date of the first vaccination, if this vaccination was with the JCOVDEN vaccine or was preceded by an infection).

The basic immunisation rate according to the DIM vaccination monitoring, when matched to the study and assuming 100% completeness of the DIM, is 80.6%. The observed rate in our study was thus 12 percentage points higher, with marked differences between the age groups: for 18- to 29-year-olds, the difference was 19 percentage points, for people ≥ 70 years only around five percentage points (Supplementary Table S7). When one assumes a certain degree of incompleteness of the DIM, say 97% completeness, the difference between the national surveillance data and the study results is reduced to 10 percentage points. With a completeness of 95%, the difference is only 8 percentage points.

Supplementary Figure S4 shows that most basic immunisations, both in the study and in the general population, were achieved before the start of the study. Interestingly, there is very good agreement between the study population and the DIM regarding basic immunization in the first months of the vaccination campaign (till around week 20), when strong priorisation rules were in place and vaccination was mainly offered by centrally organised vaccination centres. Vaccination by primary healthcare providers started in week 14 [9], which seems to have lead to increasing discrepancies in basic immunisation rates between the study population and the general population (as reflected in the DIM), reaching a peak around week 36. With the first information on the study in week 37 and the start of field work in week 43 [8], there is an indication of a small further increase in basic immunisation rates in the study population, which might be an effect of the invitation to the study, but this effect is not large. After the peak in week 36, the difference between the study population and the DIM decreases somewhat, and towards the end of the year, the basic immunization rate in the study is approaching a plateau, while the rate in the DIM continues to increase. This implies that basic immunisation was achieved earlier in study participants than in the population as a whole [9], and that the differences between the two data sources are not constant and may become smaller over time. These basic patterns were similar in all age groups (data not shown).

Discussion

We found higher rates of basic immunization in the study population than were reported to the COVID-19 Electronic Vaccination Coverage Monitoring database (Digitales Impfquotenmonitoring, DIM). This points to the presence of some selection bias in our study, with health-conscious individuals and persons that are in agreement with pandemic mitigation measures and vaccination being more likely to participate, leading to an overrepresentation of vaccinated individuals in the sample. The group of people with higher vaccination rates probably also comprises people that have easier access or are in closer contact to the healthcare system, be it because of higher education or living in an area with low socioeconomic deprivation [9], because of concomitant diseases or of working in the health systems, all factors which often coincide with a higher probability of taking part in a health survey. In the RKI-SOEP-2 study, the requirement of self-sampling capillary blood may have exacerbated this selection bias.

In this comparison, we applied some simple global corrections for certain causes of incompleteness in the DIM data that could roughly be quantified. Other causes were not quantified and instead handled by summary assumptions regarding completeness of the DIM (97% and 95%). Factors contributing to incompleteness include vaccinations received abroad (0.5% in the RKI-SOEP-2 study), the exclusion of vaccination reports with incomplete or implausible data, and a general underreporting given the pandemic situation and the accompanying strain on healthcare providers, with vaccination itself being the top priority and not so much documentation.

For the present comparison, we adapted the definition of basic immunisation to the definition in use in the DIM during 2021. While this definition was in theory identical in the analyses of the study data and the DIM data presented here, in practice one must recognize that the healthcare providers documenting the vaccinations may not always have adhered completely to this definition and mistakenly report a vaccination after a previous infection or a vaccination with the JCOVDEN vaccine as a first vaccination, not a vaccination leading to basic immunisation. Therefore, the DIM data will underestimate the prevalence of basic immunisation somewhat. The extent of this underestimation is unknown.

The differences between the prevalence of basic immunisation observed in the study and the DIM were strongest and started earliest in the youngest age group (18-29 years). This might be caused by a greater heterogeneity regarding health status in younger age groups, where most people are healthy, while there is a relatively small subgroup with e.g. concomitant diseases or working in the health system, which were included early in the vaccination priorisation, and which at the same time are likely to show a higher willingness of participating in a health survey. Moreover, other factors may contribute to the larger difference in younger age groups. Vaccinations in older age groups included in the priorisation scheme were more often carried out in specialised

vaccination centers and mobile teams (data not shown), where more complete reporting and less coding errors may be expected than in reports by primary healthcare providers or in unconventional settings such as shopping centres. Also, vaccinations with the JCOVDEN vaccine and basic immunisation through a vaccination after previous infection, both of which might be prone to higher rates of erroneous coding and thus lower completeness of the DIM register, were more common in younger age groups (data not shown).

The constant increase in basic immunisation across age groups seen in the rates provided by the DIM vaccination monitoring seems more plausible than the less stringent increase observed in the RKI-SOEP-2 study, indicating possible participation bias in the very young and the very old age groups.

In summary, we observed higher rates of basic immunisation in the RKI-SOEP-2 study than reported by the DIM vaccination monitoring, with an overall difference of 8 to 12 percentage points. This difference varied over time and differed strongly between age groups. Reassuringly, the observed differences were smaller in the higher, more vulnerable age groups.

7.2. Comparison with mandatory COVID-19 case notification

Here, the self-reported PCR-confirmed infections in the study population, which correspond to COVID-19 cases reported in the mandatory notification system in Germany [7], are compared to this notification data in order to assess possible selection bias in the study. Supplementary Figure S5 shows the proportion with known, self-reported infection in the study (observed proportion) compared to the cumulative proportion of non-fatal cases in the German population according to notification data (expected proportion). To calculate the expected proportion in the population, the infection rate based on notified cases (number of non-fatal cases, divided by population size minus the number of fatal cases) was matched to the individual participants by participation date (minus 4 days difference to symptom onset), age group and district (similarly to the estimation of underreporting, see Supplement 4, but with a different time lag). This adjusts for changes in the cumulative proportion over time and differences between districts and age groups. The expected proportion for each group was obtained by calculating groupwise weighted averages of the individually matched infection rates.

The proportion with known infection observed in the study was 8.3% (95% CI 5.8%-11.9%) for 14 to 17 years, 8.0% (95% CI 6.4%-9.9%) for 18 to 34 years, 11.6% (95% CI 9.5%-14.0%) for 35 to 49 years, 6.5% (95% CI 5.3%-7.9%) for 50 to 64 years and 4.0% (95% CI 3.1%-5.1%) for 65 years and older. The expected proportion based on notification data was 11.1% (95% CI 10.5%-11.7%) for 14 to 17 years, 10.4% (95% CI 10.1%-10.6%) for 18 to 34 years, 9.4% (95% CI 9.1%-9.7%) for 35 to 49 years, 6.5% (95% CI 6.4%-6.7%) for 50 to 64 years and 3.9% (95% CI 3.8%-4.0%) for 65 years and older. In total, the observed proportion was 7.4% (95% CI 6.6%-8.3%), only slightly lower than the expected proportion of 7.7% (95% CI 7.6%-7.9%).

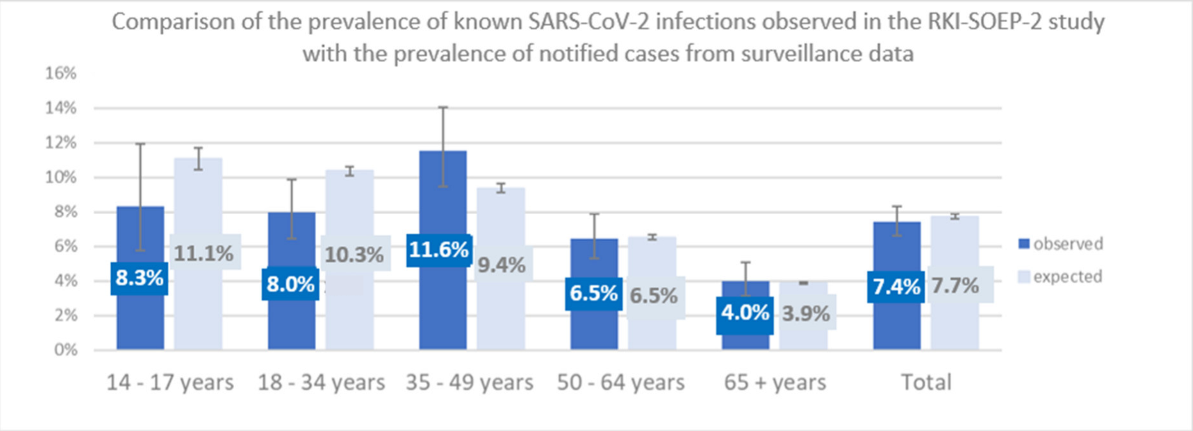

Supplementary Figure S5. Comparison of prevalence of known SARS-CoV-2 infections estimated from RKI-SOEP-2 study (observed) with prevalence of notified cases from surveillance data (expected).

In the total and in the higher age groups (50 years and older), there was good agreement between the observed and expected proportion of known infections. In the youngest two age groups (up to 34 years), however, participants were less likely to report a known infection than the general population, while in the age group 35 to 49 years the proportion observed in the study was higher than expected in the population. A possible explanation for the lower observed infection rate in the youngest age groups might be that teenagers and young

adults are generally healthy and not that much inclined to take part in a health study, whereas the subgroup of young people with health problems might be more interested in participating in the study, while at the same time having protected themselves more against infection than their peers since they were more vulnerable. Older age groups, in contrast, had higher response rates in our study [8], and at the same time a higher proportion of vulnerable individuals, so there is less potential for selection bias.

This analysis suggests that, as a whole, our sample is similar to the general population with regard to known infections, however, there is some selective participation in the three younger age groups. Infection rates and underreporting may therefore be underestimated in adolescents and young adults, and comparisons between the age groups 18-34 years and 35-49 years are uncertain, also because of different results for infection-induced seroprevalence than for infection status (see main text).

References

[1] Robert Koch-Institut. COVID-19-Impfungen in Deutschland. Zenodo. 2023.

[2] Steffen A, Rieck T, Fischer C, Siedler A. Inanspruchnahme der COVID-19-Impfung - Eine Sonderauswertung mit Daten bis Dezember 2021. Epidemiol Bull. 2022;27:3-12.

[3] Perumal N, Steffen A, Ullrich A, Siedler A. Impact of COVID-19 immunisation on COVID-19 incidence, hospitalisations, and deaths by age group in Germany from December 2020 to October 2021. Vaccine. 2022;40(21):2910-4.

[4] Perumal N, Schönfeld V, Wichmann O. Application of the screening method for estimating COVID-19 vaccine effectiveness using routine surveillance data: Germany's experience during the COVID-19 pandemic, July 2021 to March 2023. Euro Surveill. 2024; 29: 2300329.

[5] Federal Statistical Office. Population by age group. https://www.destatis.de/DE/Themen/Gesellschaft-Umwelt/Bevoelkerung/Bevoelkerungsstand/Tabellen/bevoelkerung-altersgruppen-deutschland-basis-2022.html and https://www.destatis.de/DE/Themen/Gesellschaft-Umwelt/Bevoelkerung/Bevoelkerungsstand/Tabellen/bevoelkerung-altersgruppen-deutschland.html, accessed on 11 December 2024.

[6] Robert Koch-Institut. Krebs in Deutschland für 2019/2020 [Cancer in Germany for 2019/2020]. Methods. Berlin, 2023. https://www.krebsdaten.de/Krebs/DE/Content/Publikationen/Krebs_in_Deutschland/krebs_in_deutschland_inhalt.html, accessed on 11 December 2024.

[7] Robert Koch-Institut. SARS-CoV-2 Infektionen in Deutschland. Zenodo. 2023.

[8] Bartig S, Brücker H, Butschalowsky H, et al. Corona Monitoring Nationwide (RKI-SOEP-2): Seroepidemiological Study on the Spread of SARS-CoV-2 Across Germany. Jahrbücher für Nationalökonomie und Statistik. 2022.

[9] Reis M, Michalski N, Bartig S, Wulkotte E, Poethko-Müller C, Graeber D, Rosario AS, Hövener C, Hoebel J. Reconsidering inequalities in COVID-19 vaccine uptake in Germany: a spatiotemporal analysis combining individual educational level and area-level socioeconomic deprivation. Sci Rep. 2024;14:23904.

8. Stratified analysis of the components of infection status

Supplementary Table S8 is an addition to Table 2 in the main text and presents a stratified analysis of the three components of the outcome variable ‘infection status’: (i) prior PCR-test confirmed SARS-CoV-2 infection (self-report), (ii) SARS-CoV-2 anti-nucleocapsid (anti-N) antibodies, and (iii) in non-vaccinated participants only, SARS-CoV-2 anti-spike (anti-S) antibodies. Altogether, the number of participants with information on at least one of these components was 11,154, with 1,193 participants showing evidence of prior SARS-CoV-2 infection in at least one of the components.

	Participants								
	Number with		% positive (PCR+)	Number with		% positive (N+)	Number with		% positive (S+ and non-vaccinated)
	PCR+ [a]	Available self-report on prior PCR-positive infection		N+ [a]	Valid anti-N test		S+ and non-vaccinated	Valid anti-S test and known vaccination status	
**14–99-year-olds**	922	10,889	7.4	621	10,342	6.0	164	10,501	1.6
**18–99-year-olds**	850	10,207	7.3	568	9,729	5.8	129	9,879	1.1
**Women**	504	5,874	7.0	331	5,603	5.3	89	5,683	1.3
**Men**	418	5,015	7.9	290	4,739	6.7	75	4,818	1.9
**14–17 years**	72	682	8.3	53	613	8.2	35	622	6.9
**18–34 years**	189	1,875	8.0	113	1,814	6.2	29	1,824	1.4
**35–49 years**	265	2,405	11.5	163	2,350	7.9	45	2,327	2.0
**50–64 years**	275	3,434	6.5	177	3,293	4.5	48	3,348	1.1
**65–99 years**	121	2,493	4.0	115	2,272	4.7	7	2,380	0.1
**Low deprivation[b]**	203	2,644	7.4	140	2,502	6.3	38	2,544	1.9
**Medium deprivation[b]**	590	6,621	7.7	381	6,306	5.6	106	6,406	1.5
**High deprivation[b]**	120	1,526	5.9	95	1,438	6.9	19	1,455	1.1
**Northern region**	94	1,980	4.3	77	1,910	3.8	9	1,923	0.5
**Western region**	255	3,419	7.0	165	3,252	5.3	28	3,298	0.8
**Southern region**	271	3,088	7.4	177	2,908	6.4	59	2,958	2.2
**Eastern region**	302	2,402	11.3	202	2,272	8.8	68	2,322	3.1

Supplementary Table S8. Study participants categorised according to respective information relevant for infection status estimation with, among each category, the proportion of participants showing evidence of past infection, stratified across demographic and socio-geographic characteristics, Germany (RKI-SOEP-2 study), autumn/winter 2021/22[a] (n = 11,154 participants).

All percentages are population-weighted and without correction for test characteristics and antibody waning.
All analyses for 14-99 years unless otherwise specified.
PCR+: self-reported PCR-positive infection; N+: anti-N seropositive; S+: anti-S seropositive; RKI-SOEP-2: second Corona Monitoring Nationwide study based on a population sample embedded in a dynamic cohort (the Socio-Economic Panel; SOEP); SARS-CoV-2: severe acute respiratory coronavirus 2.
[a] The RKI-SOEP-2 study collected data between 13 November 2021 and 6 March 2022 but these were predominantly obtained between November 2021 and January 2022.
[b] Numbers do not add up to total due to missing values in the variable for socioeconomic deprivation (available-case analysis).

9. Flowchart of the study population

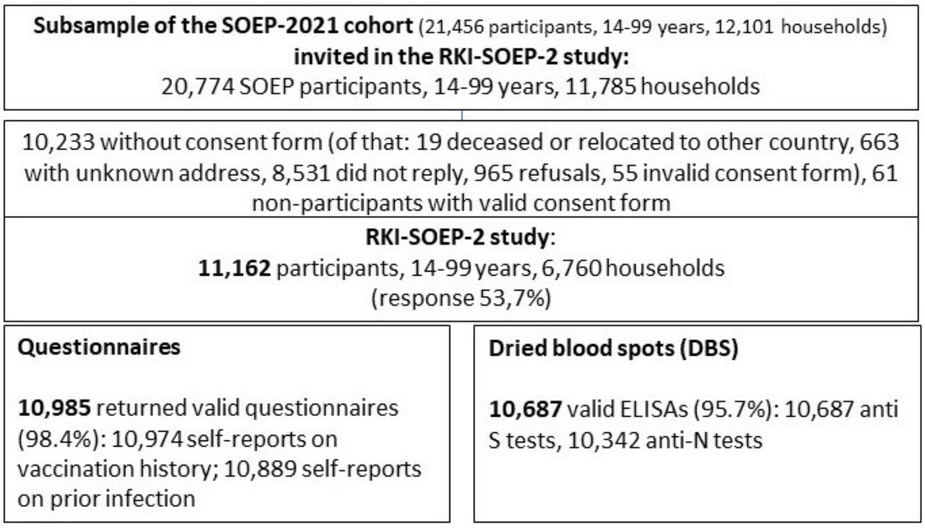

Supplementary Figure S6. Flowchart of the study population. Percentages are unweighted.

10. Seroprevalence studies in other European countries

Supplementary Table S9 gives an overview of nationwide population-based SARS-CoV-2 seroprevalence studies in other European countries other than Germany at a similar time to the RKI-SOEP-2 study. Anti-N seroprevalence is not listed here due to high heterogeneity of the sensitivity of the assays used, and the large increase of infections during the Omicron wave, so that reported prevalences are only comparable within a small time frame.

Country	Time frame	Seroprevalence (95% CI) [a]	Age (yrs)	Sample	Laboratory assay	Correction for test characteristics
**Portugal**[1]	28.09. - 19.11.2021	1+ years: 86.4% (85.2-87.6%)<br>10+ years: 92.2% [b]<br>20+ years: 94.1% [b]	1+	Patients coming for venous puncture	anti-S: Abbott SARS-CoV-2 IgG II Quant, anti-N: Abbott SARS-CoV-2 IgG I	No
**Estonia**[2]	09. - 21.11.2022	89.9% (88.5-91.2%)	18+	General population	?	?
**Finland**[3,4]	Oct to Dec 2021	around 90% [3]<br>91% (75-97%) [4]	18-69<br>18-70	General population[4]	in-house fluorescent multiplex immunoassay (FMIA)	No, study-internal Sens 99% and Spec 100%.[4]
**Scotland**[5]	20.12.2021 - 23.01.2022	91.5% (90.5-93.0%)	0+	Private households	see UK (?)	No
**Belgium**[6]	24.01. - 20.02.2022	91.3% (87.1-95.5%) [c]	18+	Private households	anti-RBD: WANTAI SARS-CoV-2 IgG ELISA customized for saliva	Yes, study-internal Sens 95.1% and Spec 96.7%. [b]
**Ireland**[7]	30.01. - 19.02.2022	96.5% (95.2-97.5%)	18+	Residual serum/plasma	anti-S: Abbott SARS-CoV-2 IgG II Quant	Yes, manufacturer-supplied Sens 98.8% and Spec 99.55%.
**UK**[8]	14. - 20.02.2022	England: 98.4% (98.1-98.6%)<br>Wales: 98.3% (97.9-98.7%)<br>Scotland: 98.3% (97.8-98.6%)<br>N. Ireland: 98.1% (97.1-98.7%)	18+	Private households	anti-S: in-house, later marketed as OmniPATH Combi SARS-CoV-2 IgG Spike ELISA[9]	No

Supplementary Table S9. Results of nationwide SARS-CoV-2 seroprevalence studies in Europe. Sens = sensitivity, Spec = specificity.

[a] Anti-S seroprevalence or (anti-S or anti-N) seroprevalence.
[b] Belgium: Anti-RBD seroprevalence. Unclear whether sensitivity and specificity only relate to the comparison of saliva vs. blood sample.
[c] Calculated from age group-specific estimates.

References

[1] Instituto Nacional de Saúde Doutor Ricardo Jorge. Relatório de Apresentação dos Resultados Inquérito Serológico Nacional COVID-19 (3ª fase). 2021. http://repositorio.insa.pt/handle/10400.18/7828, accessed 23.11.2023.

[2] University of Tartu. https://ut.ee/en/node/112720 and https://ut.ee/en/content/study-prevalence-coronavirus-estonia, access 24.11.2023.

[3] Finnish Institute for Health and Welfare. https://www.thl.fi/roko/cov-vaestoserologia/sero_report_weekly_en.html, access 28.11.2023.

[4] Solastie A, Nieminen T, Ekström N, Nohynek H, Lehtonen L, Palmu AA, Melin M. Changes in SARS-CoV-2 seroprevalence and population immunity in Finland, 2020-2022. Emerg Microbes Infect. 2023.

[5] Public Health Scotland. https://publichealthscotland.scot/repository/enhanced-surveillance-of-covid-19-in-scotland-population-based-seroprevalence-surveillance-23-february-2022/, accessed 22.11.2023.

[6] Van der Heyden J, Leclercq V, Duysburgh E et al. Prevalence of SARS-CoV-2 antibodies and associated factors in the adult population of Belgium: a general population cohort study between March 2021 and April 2022. PREPRINT Version 1. Research Square. 2023.

[7] Health Protection Surveillance Centre. Seroprevalence of antibodies to SARS-CoV-2 in adults, May 2022. http://www.hpsc.ie/a-z/nationalserosurveillanceprogramme/reports/NSP_combined_adult_paeds_cycle_1_report_final.pdf, accessed 23.11.2023.

[8] Office for National Statistics. https://www.ons.gov.uk/peoplepopulationandcommunity/healthandsocialcare/conditionsanddiseases/articles/coronaviruscovid19latestinsights/antibodies, accessed 22.11.2023.

[9] COVID-19 Infection Survey, Study Protocol Version 16.0, March 2022. https://www.ndm.ox.ac.uk/covid-19/covid-19-infection-survey/protocol-and-information-sheets, accessed 07.12.2023.

11. Results stratified by region and federal state

Here, we present a map of Germany showing the federal states and the regions used for the analyses in the main text, as well as a table with the corresponding population sizes. Further, we present analyses of combined seroprevalence, SARS-CoV-2 infection status and basic immunisation stratified by federal state.

Region	Federal state	Population size (14 years and older) per federal state	%	Population size (14 years and older) per region	%
**Northern region**	Schleswig-Holstein	2,554,575	3.5	13,139,092	18%
	Hamburg	1,601,487	2.2		
	Lower Saxony	6,978,261	9.6		
	Bremen	587,238	0.8		
	Mecklenburg-Pomerania	1,417,531	2.0		
**Western region**	North Rhine-Westphalia	15,553,739	21.5	25,457,610	35%
	Hesse	5,460,335	7.5		
	Rhineland-Palatinate	3,575,977	4.9		
	Saarland	867,559	1.2		
**Southern region**	Baden-Württemberg	9,640,638	13.3	21,088,577	29%
	Bavaria	11,447,939	15.8		
**Eastern region**	Berlin	3,180,579	4.4	12,699,778	18%
	Brandenburg	2,217,780	3.1		
	Saxony	3,531,262	4.9		
	Saxony-Anhalt	1,915,040	2.7		
	Thuringia	1,855,117	2.6		

Supplementary Table S10. Population size (14 years and older) in German federal states and the regions used for the stratified analyses in the main text, as of 31 Dec 2021.

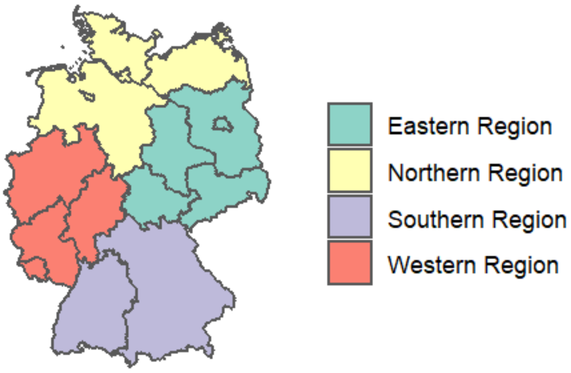

Supplementary Figure S7. Federal states in Germany, grouped in four regions for the present analysis, reflecting the geography as well as the pattern of notified infections over the course of the pandemic.

The following tables show analyses stratified by federal state. For these analyses, the sampling weights were additionally adjusted to the age distribution within each federal state. Note that this means that the age distribution for the federal state in the study corresponds to the age distribution in the population, but not that the federal states have been standardized to a common age distribution.

Results for infection-induced seroprevalence and the underreporting factor are not shown due to small sample size. As estimate of the infection rate, we can only show the variable 'infection status', which combines self-reported, known infections with uncorrected antibody results and is not corrected for test characteristics and antibody waning.

Also, we do not show the logistic regression analysis for combined seroprevalence with correction for test characteristics and antibody waning, because it produced large confidence intervals in the bootstrap analysis.

After controlling for multiple comparisons (due to the high number of 16 states being compared), we observed a lower combined seroprevalence and basic immunisation rate in Saxony and Brandenburg (combined seroprevalence: 89.1% and 87.4% vs. 95.2% overall, with correction for specificity and stratum-specific sensitivity; basic immunisation: 81.4% and 80.5% vs. 90.7% overall) and a higher infection rate in Saxony (18.5% vs. 9.8% overall). The difference in the prevalence of basic immunisation was somewhat smaller in the model-adjusted analysis (basic immunisation: 82.4% and 80.9% vs. 90.7% overall).

	Total	N+[a]	S+[a]	Combined Seroprevalence (N+ or S+), uncorrected				Combined Seroprevalence (N+ or S+), corrected for test characteristics and antibody waning
	N (column %)	%[b]	%[b]	N positive	%[b] (95% CI)	*p*-value (each state vs. the others)[d]	*p*-value (each state vs. the others), corrected for multiple testing[d]	Prevalence[c], % (95% CI)
All (total 14-99 years)	10687	6.0	90.4	9795	91.1 (90.1-91.9)			95.2 (94.0-96.3)
**Federal State**					*p = 0.0008*			
Schleswig-Holstein	423 (3.8)	4.1	92.7	395	92.8 (88.3-95.7)	0.38	1.00	95.2 (90.9-99.5)
Hamburg	238 (2.2)	7.7	90.7	223	[e]	[e]	[e]	[e]
Lower Saxony	1005 (9.5)	3.7	90.4	936	90.5 (86.7-93.3)	0.72	1.00	96.9 (92.0-101.7)
Bremen[e]	67 (0.8)	[e]	[e]	[e]	[e]	[e]	[e]	[e]
North Rhine-Westphalia	2143 (21.2)	4.6	93.1	2016	93.2 (91.2-94.8)	0.019	0.26	96.9 (94.6-99.2)
Hesse	733 (7.8)	6.9	91.3	676	91.7 (87.9-94.3)	0.71	1.00	96.9 (92.7-101.1)
Rhineland-Palatinate	383 (4.1)	4.6	89.7	352	89.7 (83.3-93.9)	0.58	1.00	95.6 (88.5-102.6)
Baden-Württemberg	1322 (13.8)	5.6	91.8	1217	91.9 (88.9-94.1)	0.50	1.00	95.8 (92.5-99.1)
Bavaria	1679 (15.7)	6.4	91.8	1546	92.8 (90.8-94.4)	0.065	0.77	96.0 (93.8-98.2)
Saarland[e]	109 (1.5)	[e]	[e]	[e]	[e]	[e]	[e]	[e]
Berlin	514 (5.0)	5.0	91.7	477	91.6 (86.5-94.9)	0.79	1.00	93.1 (88.8-97.4)
Brandenburg	406 (2.5)	9.0	83.3	357	83.8 (77.5-88.6)	0.0008	0.012	87.4 (80.9-93.9)
Mecklenburg-Pomerania	227 (1.6)	3.2	85.5	196	85.5 (76.8-91.3)	0.057	0.75	89.5 (81.3-97.7)
Saxony	726 (5.3)	12.7	83.0	621	83.3 (78.0-87.5)	<.0001	0.0003	89.1 (83.3-94.8)
Saxony-Anhalt	334 (2.6)	8.7	87.4	298	88.8 (82.9-92.8)	0.31	1.00	92.5 (86.6-98.3)
Thuringia	378 (2.7)	7.1	88.0	322	88.1 (82.7-92.0)	0.16	1.00	92.0 (86.2-97.8)

Supplementary Table S11. Combined IgG seroprevalence by federal state, in community-dwelling persons (≥14 years) in Germany (10,687 RKI-SOEP-2 study participants with valid dried blood spot specimens, sampled predominantly between November 2021-January 2022).

All percentages are population-weighted (weights additionally adjusted to the age distribution within federal states; not age-standardized).

[a] N+, anti-N seropositive. S+, anti-S seropositive. Number of available cases for anti-N-antibodies result: 10,342 (missing: 820); anti-S-antibodies result: 10,687 (missing: 475).

[b] Percentage without correction for test characteristics and antibody waning.

[c] Seroprevalence for N+ and/or S+, corrected for test characteristics (anti-S specificity = 0.994) and stratum-specific antibody waning (see Supplement 5.1 and 6.1).

[d] *p*-value for comparing each state with the other states (based on the unadjusted prevalence estimate), corrected for multiple testing (16 states) using the Bonferroni-Holm method [1].

[e] Results not shown due to small sample size.

	Total	PCR+ (self-reports) [a]	N+ [a]	unvaccinated & S+ [a]	Past SARS-CoV-2 Infection (self-reports and uncorrected antibody results)					
	**N (column %)**	**%**	**%**	**%**	**N positive**	**% [b] (95% CI)**	***p*-value (each state vs. the others) [c]**	***p*-value (each state vs. the others), corrected for multiple testing [c]**	**OR and *p*-value, adjusted [d] (95% CI)**	**Model-adjusted [d] prevalence, % (95% CI)**
All (total 14-99 years)	11154	7.3	5.9	1.3	1193	9.8 (8.9-10.7)				9.7 (8.8-10.6)
**Federal State**									*p < 0.0001*	
Schleswig-Holstein	436 (3.7)	5.1	4.1	0.1	29	6.7 (3.4-12.6)	0.237	1.00	0.88 (0.41-1.89)	7.3 (3.8-13.8)
Hamburg	248 (2.3)	4.8	7.7	0.0	18	9.4 (4.9-17.4)	0.903	1.00	1.15 (0.52-2.55)	9.3 (4.8-17.3)
Lower Saxony	1042 (9.4)	4.9	3.7	0.9	71	6.7 (4.4-10.1)	0.056	0.73	0.82 (0.49-1.35)	6.8 (4.5-10.2)
Bremen [e]	71 (0.8)	[e]	[e]	[e]	[e]	[e]	[e]	[e]	[e]	[e]
North Rhine-Westphalia	2245 (21.4)	6.6	4.6	0.7	205	8.3 (6.7-10.4)	0.11	1.00	Ref.	8.2 (6.5-10-3)
Hesse	754 (7.7)	8.5	6.9	0.8	77	11.1 (7.7-15.6)	0.47	1.00	1.37 (0.86-2.21)	10.9 (7.6-15.4)
Rhineland-Palatinate	410 (4.3)	5.8	4.6	0.7	39	7.0 (3.9-12.1)	0.23	1.00	0.80 (0.41-1.57)	6.7 (3.8-11.8)
Baden-Württemberg	1385 (13.7)	6.7	5.6	2.2	146	9.8 (7.4-12.9)	0.97	1.00	1.19 (0.77-1.83)	9.6 (7.1-12.7)
Bavaria	1761 (15.8)	7.6	6.4	1.4	196	9.7 (7.6-12.3)	0.97	1.00	1.18 (0.79-1.77)	9.6 (7.4-12.3)
Saarland [e]	111 (1.5)	[e]	[e]	[e]	[e]	[e]	[e]	[e]	[e]	[e]
Berlin	537 (5.0)	10.2	5.0	1.6	71	12.3 (8.7-17.1)	0.17	1.00	1.53 (0.95-2.46)	11.9 (8.3-16.9)
Brandenburg	423 (2.5)	12.1	9.0	2.8	61	15.5 (10.2-22.7)	0.026	0.37	1.97 (1.20-3.24)	14.8 (10.2-20.9)
Mecklenburg-Pomerania	233 (1.6)	4.8	3.2	0.2	11	5.8 (2.8-11.8)	0.15	1.00	0.76 (0.32-1.80)	6.4 (3.0-13.5)
Saxony	756 (5.3)	14.2	12.7	4.5	148	18.5 (14.3-23.5)	<.0001	<.0001	2.81 (1.89-4.17)	19.6 (15.2-24.9)
Saxony-Anhalt	354 (2.6)	6.3	8.7	0.2	46	12.0 (7.4-18.9)	0.39	1.00	1.57 (0.82-3.02)	12.2 (7.1-20.2)
Thuringia	388 (2.7)	7.1	7.1	2.3	61	11.0 (7.5-15.8)	0.53	1.00	1.40 (0.85-2.32)	11.1 (7.5-16.1)

Supplementary Table S12. SARS-CoV-2 infection status (uncorrected) by federal state, in community-dwelling persons (≥14 years) in Germany (11,154 RKI-SOEP-2 study participants, predominantly November 2021-January 2022). All percentages are population-weighted (weights additionally adjusted to the age distribution within federal states; not age-standardized).

[a] N+, anti-N seropositive. S+, anti-S seropositive. Number of available cases for SARS-CoV-2 infection status: 11,154 (missing: 8); for self-reported PCR test: 10,889 (missing: 273); anti-N-antibodies result: 10,342 (missing: 820); self-reported vaccination status & anti-S-antibodies result: 10,501 (missing: 661).

[b] Percentage without correction of antibody results for test characteristics and antibody waning.

[c] *p*-value for comparing each state with the other states (based on the unadjusted prevalence estimate), corrected for multiple testing (16 states) using the Bonferroni-Holm method [1].

[d] Logistic regression model without correction of antibody results for test characteristics and antibody waning. Model variables: sex, age group, district-level socio-economic deprivation, federal state, month of study participation (categorical variable). *p*-value for a Wald test of the variable 'federal state' within a survey logistic regression model.

[e] Results not shown due to small sample size.

	Total	At least two doses of vaccine (self-reported)	Hybrid immunity		Basic Immunisation (based on self-reports and uncorrected antibody results)					
			self-reports and uncorrected antibody results [a]	self-reports and antibody results, correct. for test characteristics and antibody waning [b]						
	N (column %)	% (95% CI)	% [a] (95% CI)	Prevalence [b], % (95% bootstrap CI)	N positive	Prevalence, % (95% CI)	*p*-value (each state vs. the others)	*p*-value, corrected for multiple testing [d]	OR and *p*-value, adjusted [c] (95% CI)	Model-adjusted [c] prevalence, % (95% CI)
All (total 14-99 years)	10932	88.8 (87.8-89.8)	7.5 (6.8-8.4)		9975	90.7 (89.7-91.6)				90.7 (89.7-91.6)
**Federal State**									*p* < 0.0001	
Schleswig-Holstein	431 (3.6)	90.3 (84.5-94.1)	e	e	400	90.8 (85.0-94.5)	0.97	1.00	0.72 (0.38-1.36)	91.4 (86.0-94.8)
Hamburg	241 (2.2)	88.1 (76.4-94.4)	9.5 (4.9-17.7)	e	223	e	e	1.00	e	e
Lower Saxony	1020 (9.5)	91.0 (87.2-93.8)	4.6 (3.1-6.7)	4.4 (2.4-7.2)	961	92.1 (88.4-94.7)	0.38	1.00	0.87 (0.51-1.47)	92.7 (89.2-95.1)
Bremen [e]	65 (0.8)	e	e	e	e	e	e	e	e	e
North Rhine-Westphalia	2197 (21.4)	91.1 (88.7-93.0)	7.1 (5.6-9.0)	7.7 (5.2-10.9)	2076	93.2 (91.0-94.8)	0.012	0.16	Ref.	93.6 (91.5-95.1)
Hesse	733 (7.6)	90.5 (86.8-93.3)	9.3 (6.3-13.4)	10.4 (6.1-16.9)	683	93.0 (89.7-95.4)	0.13	1.00	0.88 (0.51-1.52)	92.8 (89.4-95.2)
Rhineland-Palatinate	407 (4.3)	89.6 (82.9-93.8)	6.3 (3.4-11.4)	e	372	91.5 (85.6-95.1)	0.73	1.00	0.75 (0.38-1.48)	91.7 (85.9-95.2)
Baden-Württemberg	1359 (13.7)	89.4 (86.1-92.0)	6.7 (4.8-9.4)	e	1234	90.4 (87.1-92.9)	0.84	1.00	0.57 (0.34-0.95)	89.4 (85.4-92.5)
Bavaria	1731 (15.8)	90.2 (87.7-92.2)	6.9 (5.2-9.1)	7.1 (4.9-9.7)	1587	91.4 (89.0-93.3)	0.49	1.00	0.61 (0.39-0.95)	90.0 (87.1-92.4)
Saarland [e]	108 (1.4)	e	e	e	e	e	e	e	e	e
Berlin	527 (5.0)	88.1 (82.2-92.2)	10.7 (7.5-15.1)	e	497	92.0 (86.6-95.3)	0.57	1.00	0.91 (0.47-1.78)	93.0 (88.2-96.0)
Brandenburg	411 (2.5)	78.5 (70.9-84.5)	9.5 (6.1-14.6)	10.2 (5.9-16.4)	352	80.5 (72.8-86.4)	<.0001	0.0008	0.28 (0.16-0.48)	80.9 (73.4-86.7)
Mecklenburg-Pomerania	230 (1.6)	88.8 (81.5-93.5)	5.7 (2.7-11.8)	e	206	90.3 (83.1-94.6)	0.88	1.00	0.70 (0.33-1.49)	91.1 (83.7-95.3)
Saxony	745 (5.3)	77.0 (71.2-82.0)	12.4 (9.1-16.6)	13.5 (9.4-18.5)	608	81.4 (75.9-85.9)	<.0001	<.0001	0.31 (0.19-0.49)	82.4 (77.3-86.6)
Saxony-Anhalt	344 (2.6)	85.0 (78.0-90.1)	10.6 (6.2-17.5)	e	302	87.0 (80.2-91.7)	0.14	1.00	0.49 (0.25-0.95)	88.0 (80.2-93.0)
Thuringia	383 (2.7)	87.1 (81.7-91.1)	8.2 (5.2-12.8)	8.8 (4.7-13.7)	315	88.1 (82.8-91.9)	0.20	1.00	0.48 (0.27-0.83)	87.7 (81.9-91.8)

Supplementary Table S13. Basic immunisation status by federal state, in community-dwelling persons (≥14 years) in Germany (10,932 RKI-SOEP-2 study participants, predominantly November 2021-January 2022). All percentages are population-weighted (weights additionally adjusted to the age distribution within federal states; not age-standardized).

Number of available cases for at least two doses of vaccine: 10,932 (missing: 230); hybrid immunity: 10,925 (missing: 237).

[a] Percentage with hybrid immunity, without correction of antibody results for test characteristics and antibody waning.

[b] Prevalence of hybrid immunity, with correction of anti-N antibody results in vaccinated participants for anti-N specificity = 0.993 and antibody waning (see Supplement 6.3).

[c] Odds ratio and model-adjusted prevalence mutually adjusted for the variables in the table and month of participation (categorical variable). Exponentiated Wald CIs and p-value for Wald test of each variable in survey logistic regression.

[d] *p*-value for comparing each state with the other states (based on the unadjusted prevalence estimate), corrected for multiple testing (16 states) using the Bonferroni-Holm method [1].

[e] Results not shown due to small sample size.

Reference: Holm S. A simple sequentially rejective multiple test procedure. Scandinavian Journal of Statistics. 1979;6:65-70
